# Polymerization degree-driven impact on digestive enzyme inhibition by proanthocyanidins: mechanistic insights

**DOI:** 10.1016/j.fochx.2026.104465

**Published:** 2026-09-25

**Authors:** Chen Wang, Qian Gao, Zhao Le, Peng Dai, Li Ma, Shuting Zhang, Baoshan Sun

**Affiliations:** a School of Functional Food and Wine, Shenyang Pharmaceutical University, 110016 Shenyang, China; b Polo de Inovação de Dois Portos, Instituto Nacional de Investigação Agrária e Veterinária, I.P., Quinta da Almoinha, 2565–191 Dois Portos, Portugal

**Keywords:** Proanthocyanidins, Degree of polymerization, Digestive enzymes, Interaction, Inhibitory mechanism

## Abstract

This work aimed to investigate the inhibitory mechanisms of proanthocyanidins (PAs) with different polymerization degrees (DP) against pepsin, trypsin, lipase, and *α*-amylase, using enzyme inhibition and kinetic assays, turbidity, fluorescence, FTIR, CD, and SEM analyses. Results showed a significant positive correlation between mDP ranging from 1 to 12 and the inhibitory efficacy against the four enzymes, with the potency order being trypsin ≈ lipase ≈ *α*-amylase > pepsin. PA-enzyme interactions exhibited multi-site binding mediated by electrostatic interactions, with mixed-type inhibition for trypsin and non-classical inhibition for the others. Importantly, an enzyme-specific DP-related threshold phenomenon was observed. Efficient fluorescence quenching occurred when mDP exceeded 4, 2, 3, and 2.5 for pepsin, trypsin, lipase, and *α*-amylase, respectively, whereas *λ*_max_ blue shifts required higher thresholds (4–12.5). Furthermore, high-DP (mDP 12) formed more compact PA-enzyme complexes than low-DP (mDP 2). This study provides mechanistic insights into the enzyme-specific inhibitory differences between low-DP and highly polymerized PAs.

## Highlights

• Mechanisms study in the interaction of PAs with DP by four digestive enzymes.
• A consistent DP correlation was revealed for inhibition of four digestive enzymes.
• Comprehensive assessment in binding mode and constant of PAs with DP to four digestive enzymes.
• Secondary structure analysis of four digestive enzymes to evaluate the effects of PAs with DP.
• Visual analysis first found PA-enzyme complex compaction with increasing DP.

## 1 Introduction

Digestive enzymes mediate the hydrolytic catabolism of dietary proteins, lipids, and starches, thereby governing nutrient bioavailability and sustaining metabolic homeostasis (Hyder et al., 2023; Saha & Pathak, 2021). Dysregulated enzymatic activity disrupts nutrient absorption and is closely associated with the pathogenesis of metabolic complications, including obesity, type 2 diabetes mellitus, and metabolic syndrome (Chew et al., 2023; James, 2018). The targeted inhibition of gastrointestinal digestive enzymes has emerged as a feasible nutritional strategy for alleviating diet-induced metabolic abnormalities (Kalra et al., 2021; Xu et al., 2024). Although synthetic enzyme inhibitors enable effective postprandial metabolic regulation, their clinical application is frequently compromised by adverse gastrointestinal reactions such as bloating and diarrhea (Barrientos-Ávalos et al., 2024; DiNicolantonio et al., 2015). In this regard, naturally occurring plant bioactives are considered well-tolerated alternatives for developing novel functional foods and nutraceuticals.

Proanthocyanidins (PAs), a category of oligomeric and polymeric flavan-3-ols, are prevalent dietary polyphenols found abundantly in plant-derived foods (Favari et al., 2020; Ribas-Agustí et al., 2018). Owing to their limited systemic bioavailability, PAs primarily exert their biological effects within the gastrointestinal lumen, with digestive enzyme inhibition being the most extensively documented function (Ćorković et al., 2022; Hao et al., 2025; Martinez-Gonzalez et al., 2017; Singh et al., 2022; Xu et al., 2021a; Zhu & Oteiza, 2024). Current research on the enzyme-inhibitory potential of PAs predominantly relies on plant-derived PA samples with varying degrees of purification, which can be hierarchically categorized into crude extracts, partially purified mixtures, and structurally defined monomers. Regarding crude PA extracts, various raw plant materials have been validated to exhibit reliable digestive enzyme inhibitory activity. For example, crude PA extracts from Chinese quince fruit demonstrate dose-dependent inhibition of *α*-amylase (Kong et al., 2022), while extracts from Cinnamomum longepaniculatum leaves, walnut green husks, cocoa, grape seed, and berries (e.g., *Aronia melanocarpa*) show dose-responsive dual inhibitory effects on pancreatic *α*-amylase and lipase (Buchholz & Melzig, 2015; Liu et al., 2017; Qi et al., 2025; Sosnowska, Kajszczak & Podsędek, 2022a). Furthermore, crude PA-rich samples from diverse plant sources exhibit broad-spectrum enzyme inhibition. Specifically, PAs extracted from Anogeissus pendula and *Eugenia jambolana* leaves modulate serine protease activity to enhance protein utilization (Singh et al., 2022).

In comparison to unprocessed crude extracts, partially purified or fractionated PA mixtures have been utilized in more refined studies, with research priorities focusing on carbohydrate-digestive enzymes. Purified PAs from *Crataegus pinnatifida Bge.* have been shown to efficiently inhibit *α*-glucosidase (Liang et al., 2022), whereas condensed tannin fractions have been employed to investigate the kinetic inhibition mechanism of *α*-amylase (Kato et al., 2017). Bayberry leaves are frequently used as a research model for purified PA mixtures. Successive studies have demonstrated that purified PAs derived from bayberry leaves exert significant inhibitory effects on both *α*-amylase (mixed-type kinetic inhibition) and *α*-glucosidase. The digestive regulatory efficacy of these compounds has been further validated using *Drosophila melanogaster* in vivo models (Wang et al., 2019; Wang et al., 2020; Wang et al., 2022). In contrast to the extensive studies on mixed PA samples, research on structurally defined individual PA components is limited, focusing primarily on well-characterized dimers and trimers of PAs. As a typical dimeric PA, procyanidin B2 exhibits notable *α*-glucosidase inhibition through a mixed-type inhibitory mechanism, providing direct molecular-level evidence for the enzyme-suppressive activity of single PA units (Han et al., 2018).

Despite the well-documented enzyme-inhibitory potency of PAs in previous studies, inherent limitations persist in the current research frameworks. Complex endogenous impurities in crude and partially purified extracts significantly hinder the identification of intrinsic PA structure-activity relationships. The degree of polymerization (DP) is widely acknowledged as a fundamental structural parameter influencing PA bioactivity (Chen et al., 2024; Mannino et al., 2021); existing DP-related investigations often rely on discontinuous DP gradients. Furthermore, current research exhibits a clear enzyme selectivity bias, with most studies concentrating on carbohydrate- and lipid-digesting enzymes while overlooking gastrointestinal proteases such as pepsin and trypsin (Li et al., 2015; Sosnowska, Podsędek & Kucharska, 2022b). Overall, these limitations have prevented a systematic clarification of whether the positive correlation between DP and inhibitory performance represents a universal regulatory characteristic of different digestive-enzyme systems.

Mechanistically, inhibitory performance is conventionally quantified using IC₅₀ values and inhibition rates reported in the literature. Kinetic analysis is occasionally performed; however, integrated evidence combining kinetic profiles, spectroscopic conformational characterization, thermodynamic signatures, and macroscopic morphological visualization remains limited (Chen et al., 2023; Sosnowska et al., 2022b). Computational simulations have proposed superior inhibitory efficiency for low-DP PAs (Vazquez-Flores et al., 2018); however, robust experimental validation remains insufficient. Furthermore, it remains unclear whether DP-mediated inhibitory differences arise from divergent binding affinities, enzyme conformational transitions, or intermolecular aggregation of PA-enzyme complexes. The lack of systematic multi-technique evidence impedes an in-depth mechanistic understanding of PA-enzyme interactions.

The objective of the present work was to systematically investigate the inhibitory mechanisms of PAs with varying DP on key digestive enzymes, including pepsin, trypsin, pancreatic lipase, and *α*-amylase. To achieve this goal, a series of purified PA fractions was isolated sequentially using column chromatography coupled with high-speed countercurrent chromatography (HSCCC). The inhibitory effects of these PA fractions on different digestive enzymes were evaluated using enzyme kinetic assays, and multi-spectroscopic analyses and scanning electron microscopy (SEM) were employed to characterize the aggregation behavior and structural features of PA fractions and PA-enzyme complexes. This study integrates macroscopic inhibitory performance with microscopic structural and interaction characteristics, providing novel mechanistic insights into PA-digestive enzyme interactions.

## 2 Materials and methods

### 2.1 Material

Grape seed proanthocyanidins (purity ≥95%) were provided by Tianjin Jianfeng Natural Product R&D Co., Ltd. (Tianjin, China). Porcine pepsin (Pep, 10 units/mg), porcine pancreatic lipase (Lip, 30 units/mg), porcine pancreatic *α*-amylase (Amy, 9.4 units/mg), bovine hemoglobin, *N*-phenylacetyl-DL-arginine-p-nitroamide hydrochloride (BAPNA), and palmitic acid *p*-nitrophenyl ester (*p*-NPP) were purchased from Shanghai Yuan Ye Biotechnology Co., Ltd. (Shanghai, China). Porcine pancreatic trypsin (Try, 10 units/mg) was purchased from Shanghai Macklin Biochemical Co., Ltd. (Shanghai, China). Soluble starch was obtained from Hengxing Chemical Reagent (Tianjin, China). Tris (hydroxymethyl) aminomethane (Tris) was purchased from Beijing Solarbio Science & Technology Co., Ltd. (Beijing, China). 3,5-Dinitrosalicylic acid (DNS) was obtained from the Tianjin Guangfu Fine Chemical Research Institute (Tianjin, China). Ninhydrin was purchased from Tianjin Ruijingtai Chemical Co., Ltd. (Tianjin, China). All other chemicals were of analytical grade and obtained from local suppliers.

### 2.2 Fractionation and purification of PAs according to their degrees of polymerization

Commercial grape seed proanthocyanidins (GSE) were first purified by C18 column chromatography (Sun et al., 1999), subsequently fractionated according to their DP using high-speed counter-current chromatography (HSCCC), based on a previous procedure (Zhang et al., 2017) with minor modifications. In brief, aqueous GSE samples were loaded onto an open C18 column (200 × 25 mm i.d., 25–40 μm particle size; Merck, Germany). Elution with water adjusted to pH 7.0 was performed to eliminate phenolic acids and other small phenolics, followed by elution with methanol to recover PAs. The methanolic fraction was lyophilized for at least 48 h. The resulting powder, corresponding to the purified PAs, was stored at −20 °C in the dark prior to HSCCC fractionation. Semi-preparative HSCCC (Tauto Biotech TBE—300B, Shanghai) was used to fractionate the purified PAs into eight fractions (F_1_-F_8_) according to their DP, using n-hexane-ethyl acetate-water (1:50:50, *v*/v/v) as the solvent system. The optimized operating conditions were as follows: head-to-tail elution mode, flow rate of 3.0 mL/min, revolution speed of 900 rpm, separation temperature of 25 °C, and sample load of 300 mg.

### 2.3 Structural characterization and purity of PAs with different DP

#### 2.3.1 Phloroglucinolysis-UPLC analysis

Phloroglucinolysis was employed to determine the structural composition and mean degree of polymerization (mDP) of each fraction (F_1_-F_8_) isolated via HSCCC. This procedure was performed according to a previously described method (Zhang et al., 2017) with a few modifications. Briefly, 6 mg of each HSCCC-isolated fraction was dissolved in 1 mL of methanol. This solution was then added to 1 mL of methanolic phloroglucinol solution (50 mg/mL) that was acidified with 0.2 M HCl in a stoppered test tube. The tube was incubated at 50 °C for 20 min to allow the phloroglucinolysis reaction to proceed, and subsequently immersed in an ice bath (0 °C) to terminate the reaction. The resulting reaction mixture was filtered through a 0.22 μm membrane filter before UPLC analysis. The UPLC chromatographic conditions were identical to those reported by Zhang et al. (Zhang et al., 2017).

#### 2.3.2 Fourier transform infrared (FTIR) spectroscopy analysis

FTIR spectra of PA fractions F_1_-F_8_ (in powder form) were recorded in the range of 400–4000 cm^−1^ using an FTIR-650 spectrometer (Tianjin Port East Technology Co., Ltd., China) equipped with a pyroelectric detector and a resolution of 4 cm^−1^.

#### 2.3.3 Zeta potential analysis

A Zetasizer NANO-ZS90 Malvern laser particle size analyzer (Zetasizer Advance, Malvern Panalytical, UK) was used to determine the zeta potentials of the sample solutions. The PA fractions F_1_-F_8_ were diluted with 0.1% (*w*/*v*) dimethyl sulfoxide (DMSO) in Milli-Q water prior to zeta potential measurement.

### 2.4 Inhibitory effects, kinetics, and inhibition type of PAs with different DP on key digestive enzymes

Pep: Pep inhibitory activity was assessed according to the literature, with some modifications (Meridor & Gedanken, 2014). Pep was dissolved in HCl solution (pH 2) to prepare an enzyme stock solution with a concentration of 3 mg/mL. Briefly, 30 μL of the enzyme stock solution, 25 μL of PAs solution, and 95 μL of HCl solution (pH 2) were thoroughly mixed in a centrifuge tube. The mixture was incubated at 37 °C with shaking for 30 min, followed by the addition of 150 μL of 0.1 mmol/L bovine hemoglobin solution (pH 2). After further incubation at 37 °C with shaking for 10 min, the enzymatic reaction was terminated by adding 300 μL of 5% (*w*/*v*) trichloroacetic acid (TCA) solution. The mixture was allowed to stand at 37 °C for 10 min and then centrifuged at 12000 *g* for 3 min. Subsequently, 400 μL of the supernatant was transferred and mixed with 150 μL of 0.4 M Na₂CO₃ solution and 350 μL of ninhydrin reagent. The mixture was heated in a boiling water bath for 5 min, after which 500 μL of distilled water was added, and the absorbance was measured at 570 nm, denoted as A_1_. The absorbance values were recorded as A_2_ and A_3_ in the absence of the enzyme and sample, respectively, while A_4_ represented the absorbance in the absence of both the enzyme and sample. An equal volume of ultra-pure water was used as a substitute for the enzyme and/or sample solution. The formula for the enzyme inhibition rate is as follows. To determine the inhibition type and enzymatic kinetic parameters, experiments were performed using serial gradients of enzyme and substrate concentrations. The assay protocols for the inhibition rate and kinetic analysis of the other tested enzymes were consistent with those described for Pep.

(1) $$\text{Enzyme inhibition rate}\%=\left(1-\frac{A_{1}-A_{2}}{A_{3}-A_{4}}\right)\times 100$$

Try: Try inhibitory activity was determined using a modified method based on previous reports (Yakoby & Raskin, 2004). Try was dissolved in 50 mM phosphate-buffered saline (PBS) buffer (pH 8.0) to prepare an enzyme stock solution with a concentration of 0.5 mg/mL. Briefly, 80 μL of the enzyme stock solution was mixed thoroughly with 80 μL of the PAs solution, followed by incubation at 37 °C with shaking for 30 min. Subsequently, 400 μL of the substrate (0.5 mg/mL, BAPNA) was added, and the mixture was incubated for 10 min. The enzymatic reaction was terminated by adding 200 μL of 33% (*w*/*v*) TCA solution, and the absorbance was measured at 420 nm.

Lip: Lip inhibitory activity was evaluated following a literature method with minor adjustments (Syedd-León et al., 2020). An analytical buffer was prepared containing 25 mM NaCl, 10 mM CaCl₂, and 100 mM Tris, and the pH was adjusted to 8.0. Briefly, 564 μL of the analytical buffer was mixed with 200 μL of 1 mg/mL Lip solution and 120 μL of the PAs solution, followed by incubation at 37 °C for 30 min. Then, 36 μL of 16 mM *p*-NPP solution was added. After thorough mixing and incubation for 20 min, 100 μL of 50% (*w*/*v*) TCA solution was added to inactivate Lip. A total of 750 μL of the reaction mixture was mixed with 120 μL of 3 M Na₂CO₃ solution, and then centrifuged at 12000 rpm for 3 min. The absorbance of the supernatant was measured at 410 nm.

Amy: Amy inhibitory activity was measured according to a documented procedure with slight modifications (Kusano et al., 2011). 1% (*w*/*v*) soluble starch solution was prepared in 100 mM phosphate buffer (pH 6.9), which was heated and stirred at 100 °C for 10 min to ensure complete dissolution. 4 mg/mL Amy solution was prepared in 100 mM phosphate buffer (pH 6.9). Briefly, 120 μL of the Amy solution was mixed thoroughly with 120 μL of the PAs solution, followed by incubation at 37 °C with shaking for 30 min. Then, 120 μL of the soluble starch solution was added to the mixture in a centrifuge tube, and the mixture was incubated at 37 °C with shaking for 15 min. The reaction was terminated by adding 200 μL of DNS reagent and heating in a boiling water bath for 5 min. After cooling slightly, 1040 μL of phosphate buffer (100 mM, pH 6.9) was added, and the absorbance of the solution was measured at 540 nm.

### 2.5 Turbidity analysis of PAs with different DP and key digestive enzymes

Turbidity measurements were performed using a previously reported method (Li et al., 2019) with minor modifications, as follows. Enzyme solutions Pep (3.0 mg/mL), Try (1.2 mg/mL), Lip (1.0 mg/mL), and Amy (2.0 mg/mL) were mixed with PA fractions F_1_-F_8_ at mass ratios of 1:3, 1:1, and 3:1. The mixtures were co-incubated at 37 °C for 30 min. The absorbance at 600 nm was recorded using a multimode microplate reader (Varioskan LUX, Thermo Fisher Scientific, USA) to characterize the solution turbidity.

### 2.6 Interaction mechanism between PAs with different DP and key digestive enzymes

#### 2.6.1 Fluorescence spectroscopy analysis

Fluorescence measurements of the samples were performed using a multimode microplate reader (Varioskan LUX, Thermo Fisher Scientific, USA). Samples were prepared by mixing fixed concentrations of Pep, Try, Lip, and Amy with gradient concentrations of PA fractions F_1_-F_8_. The final concentrations of Pep, Try, Lip, and Amy were 3, 1, 4.5, and 4 mg/mL, respectively, while the final concentrations of F_1_-F_8_ in the mixtures were 0, 0.06, 0.09, 0.12, 0.15, and 0.18 μg/mL. Fluorescence emission spectra of the digestive enzymes were recorded over a wavelength range of 300–500 nm, with an excitation wavelength of 280 nm, at temperatures of 291, 298, and 310 K. The excitation and emission slit widths were set to 5.0 nm. Background signals from the PAs and buffer solution were subtracted from the raw mixture spectra. The maximum fluorescence emission wavelengths of the digestive enzymes were used to calculate the quenching parameters and binding constants (Zhang et al., 2024).

The fluorescence quenching data were analyzed by the Stern-Volmer equation:

(2) $$\frac{F_{0}}{F}=1+K_{q}\tau_{0}\left[Q\right]=1+K_{\mathrm{sv}}\left[Q\right]$$

F and F_0_ represent the fluorescence intensities of the digestive enzymes in the presence and absence of the quencher (PAs), respectively. Where [Q] denotes the concentration of the quencher, K_q_ is the biomolecular quenching rate constant, and K_sv_ refers to the Stern-Volmer dynamic quenching constant. τ_0_ represents the average fluorescence lifetime of the biomolecule without the quencher, which is typically approximately 10^−8^ s.

Moreover, the binding constant K_a_ and number of binding sites n for PA fractions F_1_-F_8_ were calculated using Eq. (3). Static quenching ultimately reaches a thermodynamic equilibrium between free and bound molecules, which can be described using the logarithmic form of the modified Stern-Volmer equation (Zhang et al., 2024):

(3) $$\log\frac{F_{0}-F}{F}=\log K_{a}+\mathrm{nlg}\left[Q\right]$$

where the number of binding sites (n) and binding constant (K_a_) can be obtained from the slope and intercept of the double logarithmic plot of lg[(F_0_—F)/F] versus [Q].

To determine the main driving forces between PA fractions F_1_-F_8_ and the four digestive enzymes (Pep, Try, Lip, and Amy), thermodynamic parameters were calculated using the van't Hoff equation (Ge et al., 2021):

(4) $$\ln K_{a}=-\frac{∆H}{\mathrm{RT}}+\frac{∆S}{R}$$

(5) ∆G=∆H−T∆S

where R denotes the gas constant (8.314 J·mol^−1^·K^−1^), T represents the absolute temperature (291, 298, and 310 K), and ΔG is the Gibbs free energy change of the system. The enthalpy change (ΔH) and entropy change (ΔS) values were obtained from the slope and y-intercept of the linear fitting plot of lnK_a_ versus 1/T.

#### 2.6.2 Circular dichroism (CD) spectroscopy analysis

CD spectra were recorded using a CD spectrometer (Bio-Logic MOS 450, Claix, France). Far-UV CD spectra (190–250 nm) were measured using a 1.0 nm path length cuvette under a constant nitrogen flush. The concentrations of Pep, Try, Lip, and Amy were maintained at 0.6 mg/mL, with the mass ratios of fractions F_1_-F_8_ to each enzyme fixed at 1:3. All data are expressed as mean residue ellipticity [θ] (deg cm^2^ dmol^−1^). Baseline correction was performed by subtracting the CD spectrum from the buffer. The CD spectral data were analyzed using the online SELCON3 program via DichroWeb (http://dichroweb.cryst.bbk.ac.uk/html/home.shtml) (Zeng et al., 2016) to quantify the secondary structures of Pep, Try, Lip, and Amy.

#### 2.6.3 FTIR spectroscopy analysis

FTIR spectroscopy was employed to analyze the secondary structures of the samples using an FTIR-650 spectrometer (Tianjin Port East Technology Co., Ltd., China). Briefly, Pep, Try, Lip, and Amy were individually mixed with PA fractions F_1_-F_8_ at a mass ratio of 1:3. The final concentration of each enzyme (Pep, Try, Lip, and Amy) in the mixtures was maintained at 9 mg/mL. After co-incubation at 37 °C for 30 min, the mixtures were centrifuged at 12000 rpm for 1 min, and the supernatant was discarded. The resulting sample pellets were lyophilized, ground with potassium bromide (KBr) powder, and pressed into pellets for FTIR measurements. The spectra were collected over the wavelength range of 400–4000 cm^−1^.

#### 2.6.4 Scanning electron microscope (SEM) analysis

The microparticle morphologies of fractions F_2_, F_8_, F_2_-digestive enzyme complexes (Pep, Try, Lip, and Amy), and F_8_-digestive enzyme complexes (Pep, Try, Lip, and Amy) were observed and photographed using a scanning electron microscope (Zeiss Gemini Sigma 300 VP SEM, Germany) at an accelerating voltage of 5 kV, with magnifications of 200×, 500×, and 1000×. The mixing ratios of F_2_ with digestive enzymes and F_8_ with digestive enzymes were consistent with those used in the FTIR analysis. Prior to observation, all samples were mounted on sample stages using double-sided adhesive tape and sputter-coated with gold (Chen et al., 2025).

### 2.7 Statistical analysis

All experiments were performed in triplicate, and the data are presented as the mean ± standard deviation of three independent replicates. Statistical analysis was conducted using one-way analysis of variance (ANOVA) with SPSS software, and significant differences between means were identified at a significance level of *p* < 0.05. All figures were plotted using Origin and GraphPad Prism software packages.

## 3 Results and discussion

### 3.1 Fractionation and purification of PAs according to their degrees of polymerization

The fractionation of PAs according to their DP remains challenging because of their structural complexity and diversity. In the present study, grape seed PA extract was initially purified using C18 column chromatography and subsequently fractionated by HSCCC. Fig. S1 shows the typical HSCCC chromatogram of the purified PAs according to DP. Eight different fractions were successfully separated under the optimized HSCCC conditions. Phloroglucinolysis was followed by HPLC analysis of each fraction (Fig. S2), which revealed chromatograms containing exclusively peaks corresponding to the constituent units of PAs, with no detectable signals from other phenolic compounds present. These results indicate that all isolated fractions possess high PA purity.

### 3.2 Structural characterization of PAs with different DP

#### 3.2.1 Structural characterization by phloroglucinolysis

PA fractions F_1_-F_8_ with different DP were successfully isolated via HSCCC. However, elucidating the complex structural features of PAs remains an analytical challenge and is essential for understanding the structural basis underlying their biological functions. Due to their large molecular weight and structural complexity, traditional analytical methods are limited in their ability to characterize the structures of PAs. Phloroglucinolysis was employed to depolymerize PAs, enabling the identification and differentiation of terminal and extension units, as well as the determination of subunit composition and quantitative evaluation of DP (Zhang et al., 2017). Table 1 presents the structural composition data of different PA fractions isolated, that is, mDP, percentages of terminal and extension units, and percentage of galloylation (G%). The mDP values of PA fractions F_1_ to F_8_ exhibited a significant increasing trend, ranging from 1.09 to 12.24, indicating that the fraction effectively separated PAs according to their DP. For terminal units, catechin (C) and epicatechin (EC) are predominant at lower mDP values (F_1_-F_6_), whereas at higher mDP values (F_7_-F_8_), similar proportions of C, EC, and epicatechin 3-O-gallate (ECG) were observed. For extension units, EC is predominant for all PA fractions, and its proportion generally increases with increasing mDP (with the exception of F_4_), reaching 61.55% in F_8_. The percentage of extension unit ECG increases with mDP at lower values (F_1_-F_3_), then reaches the maximum and remains relatively stable at approximately 26–27% (F_5_-F_8_). Similarly, the percentage of galloylation (G%) increases with mDP at lower values (F_1_-F_3_) before plateauing at approximately 30–31%. Collectively, these results reveal a clear DP-related structural pattern in PAs, characterized by higher proportions of EC and ECG extension units at higher mDP values.

**Table 1:** Characterization and structural composition (percent in moles) of proanthocyanidin fractions isolated from HSCCC.

Fraction	Terminal Units			Extension Units			G%	mMM	mDP
	C	EC	ECG	CP	ECP	ECGP			
F_1_	49.66 ± 3.30^a^	35.07 ± 0.91^a^	6.66 ± 1.08^a^	1.36 ± 0.14^d^	5.34 ± 2.72^e^	1.90 ± 0.22^d^	8.56 ± 1.27^c^	317.54 ± 10.41^h^	1.09 ± 0.04^h^
F_2_	12.94 ± 1.10^d^	26.48 ± 0.77^b^	4.95 ± 0.86^abc^	15.71 ± 1.09^a^	31.09 ± 1.76^d^	8.83 ± 1.05^c^	13.78 ± 1.37^b^	671.72 ± 31.30^g^	2.32 ± 0.11^g^
F_3_	23.48 ± 2.27^b^	9.15 ± 1.06^c^	4.61 ± 1.37^bc^	6.26 ± 0.63^bc^	43.63 ± 0.87^c^	12.87 ± 0.32^b^	17.48 ± 1.68^b^	773.44 ± 3.34^f^	2.67 ± 0.01^f^
F_4_	17.82 ± 1.22^c^	10.53 ± 0.77^c^	4.67 ± 1.17^bc^	6.67 ± 0.41^bc^	48.12 ± 0.08^c^	12.20 ± 0.10^b^	16.87 ± 1.10^b^	878.51 ± 13.63^e^	3.03 ± 0.05^e^
F_5_	9.56 ± 0.67^e^	7.62 ± 0.22^d^	5.09 ± 0.95^abc^	6.50 ± 4.86^bc^	45.00 ± 3.98^c^	26.24 ± 1.80^a^	31.33 ± 2.75^a^	1303.08 ± 33.79^d^	4.49 ± 0.12^d^
F_6_	8.27 ± 0.41^e^	6.25 ± 1.47^d^	3.87 ± 0.05^bc^	9.05 ± 0.23^b^	45.11 ± 1.36^c^	27.45 ± 0.38^a^	31.32 ± 0.41^a^	1583.10 ± 115.83^c^	5.46 ± 0.40^c^
F_7_	4.74 ± 1.00^f^	3.34 ± 0.34^de^	5.40 ± 1.30^ab^	6.41 ± 0.47^bc^	53.71 ± 3.38^b^	26.40 ± 3.32^a^	31.80 ± 3.77^a^	2152.63 ± 64.63^b^	7.42 ± 0.22^b^
F_8_	2.97 ± 0.05^f^	1.98 ± 0.23^e^	3.22 ± 0.11^c^	2.82 ± 3.00^cd^	61.55 ± 4.99^a^	27.46 ± 2.12^a^	30.68 ± 2.23^a^	3548.92 ± 65.22^a^	12.24 ± 0.22^a^

Abbreviations: C, (+)-catechin; EC, (−)-epicatechin; ECG, (−)-epicatechin-3-O-gallate; ECP, (−)-epicatechin-phloroglucinol derivative; CP, (+)-catechin-phloroglucinol derivative; ECGP, (−)-epicatechin-3-O-galloyl-phloroglucinol derivative; mMM, mean molecular mass; mDP, mean degree of polymerization; G%, moles percentage of galloylation. Different letters in a column indicate significant differences at *p* < 0.01; Statistically, a, b, c, d, e, f, and g followed the values indicating significant differences among these values.

#### 3.2.2 FTIR analysis

FTIR spectroscopy was employed to qualitatively characterize the functional groups and molecular conformational features of PA fractions with varying DP. As depicted in Fig. 1A and Table S1, the eight purified PA fractions (F_1_-F_8_) exhibited distinguishable spectral profiles and absorption intensities. The broad stretching band of phenolic hydroxyl groups centered at 3397 cm^−1^ remained nearly invariant in position across all DP, while its absorption intensity decreased with increasing DP. Hydrogen-bonded hydroxyl vibrations are typically observed in the 3200–3400 cm^−1^ region, particularly around 3400 cm^−1^ for catechin and epicatechin with intramolecular hydrogen-bonding (Hu et al., 2010; Khedkar et al., 2010; Ricci et al., 2015). Therefore, this band was mainly attributed to the intramolecular hydrogen bonding in the PA fractions. Moreover, the reduced band intensity at higher DP was likely associated with the formation of more extensive hydrogen-bonding networks (Kim & Kim, 2003).

**Fig. 1:**
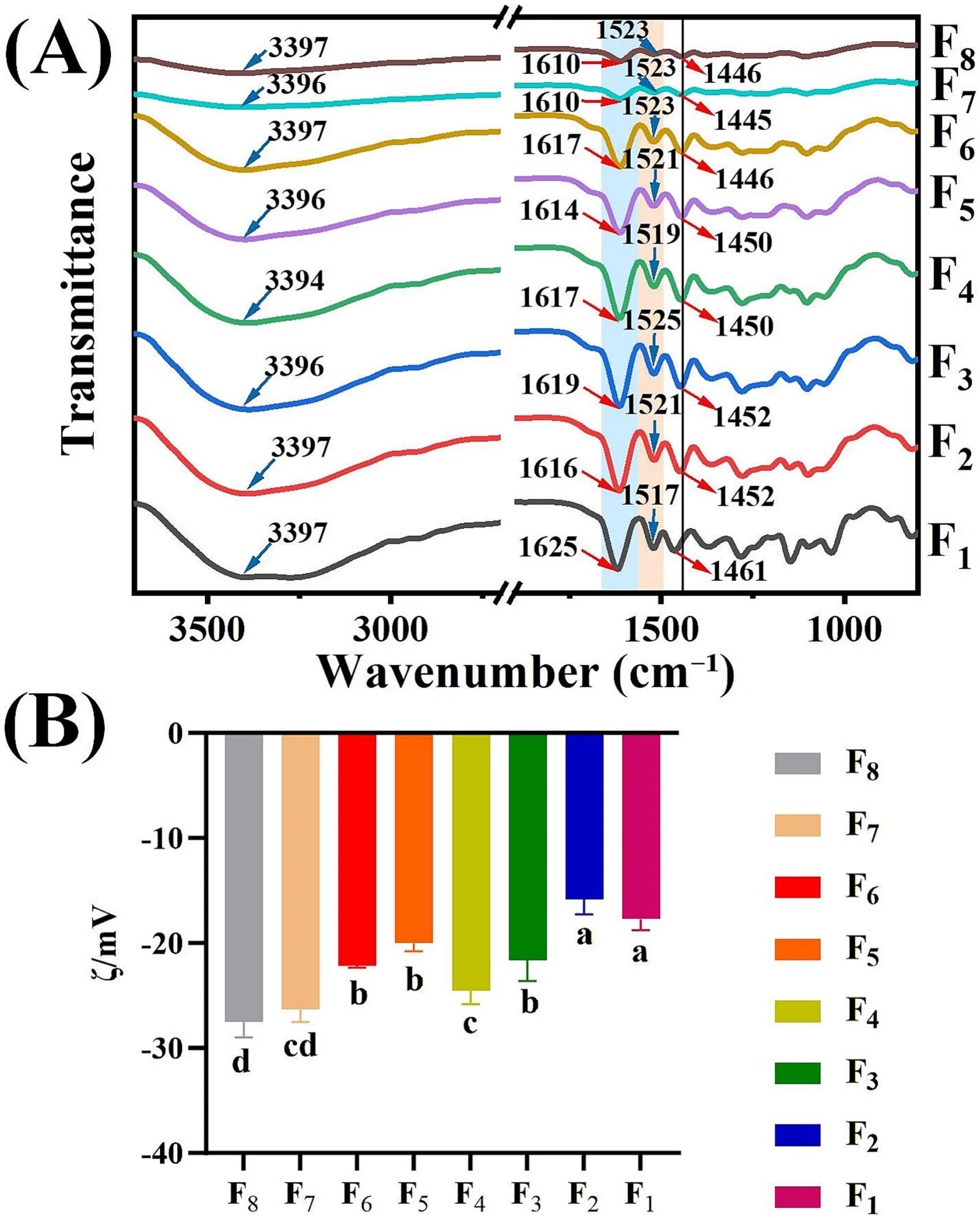
(A) FTIR spectra of proanthocyanidins with different degrees of polymerization (F_1_-F_8_). (B) Zeta potential of proanthocyanidins with different degrees of polymerization (F_1_-F_8_). Different letters indicate significant differences (*p* < 0.05).

Characteristic absorptions of aromatic and heterocyclic C=C skeletal vibrations were observed in the regions of 1610–1625, 1517–1525, and 1445–1461 cm^−1^. The band at 1517–1525 cm^−1^ corresponds to the skeletal stretching modes of the aromatic ring skeletal stretching of ortho-disubstituted aromatic B-rings, while the absorption at 1610–1625 cm^−1^ arises from the C=C-O deformation of the C-ring in the stable trans configuration (Foo, 1981; Tammer, 2004). The band at 1450 cm^−1^ is related to the in-plane vibration of the pyran heterocyclic group (Tammer, 2004). In addition, no distinct absorption band of galloyl groups appeared at approximately 1690 cm^−1^, further suggesting that non-galloylated flavan-3-ols predominated in the PA fractions. These FTIR fingerprints were in good agreement with the phloroglucinolysis results.

*Trans*-Configured flavan-3-ols are known to adopt a stable chair conformation with a sterically favorable B-ring orientation, forming a linear backbone with high structural rigidity (Rösch et al., 2004). Increased molecular rigidity restricts the vibrational amplitude, thereby reducing the IR absorption intensity (Olejar et al., 2014). This mechanism may account for the gradual attenuation of the characteristic peaks along the DP gradient. The prevalence of the *trans* conformation further indicated that PA chain elongation progressively enhanced the overall molecular rigidity with increasing DP.

#### 3.2.3 Zeta potential analysis

Zeta potential analysis was utilized to investigate the surface charge characteristics of PA fractions. As depicted in Fig. 1B, all purified PA fractions (F_1_-F_8_) demonstrated negative zeta potentials in a 0.1% aqueous DMSO solution. These values increased from 17.73 mV to 27.53 mV with an increase in mDP. The negative charge of phenolic compounds is primarily attributed to the deprotonation of phenolic hydroxyl groups (Lv et al., 2021). Higher absolute zeta potential values indicate greater hydroxyl deprotonation in PAs with higher mDP. All PA fractions exhibited similar total phenolic hydroxyl contents at equivalent mass concentrations. Theoretically, molecules with an identical number of hydroxyl groups should exhibit similar surface charges, irrespective of structural differences. However, the observed increase in absolute zeta potential with DP suggests that total hydroxyl content alone does not account for the increased deprotonation in high-DP PAs. This indicates that additional factors influence the surface charge of PAs. A higher absolute zeta potential signifies stronger electrostatic repulsion between molecules, thereby preventing aggregation (Wang et al., 2023). The variation in surface charges among PA fractions can be attributed to differing hydroxyl dissociation behaviors, which may be influenced by their self-aggregation patterns. As DP increases, electrostatic repulsion is enhanced, molecular aggregation is reduced, and deprotonation of phenolic hydroxyl groups is promoted. Consequently, high-DP PAs exhibit improved dispersion stability and stronger surface electronegativity.

Integrating all structural analyses, phloroglucinolysis demonstrated a progressive increase in the mDP from F_1_ to F_8_. FTIR spectroscopy revealed rigid and linear molecular configurations in high-DP fractions, while zeta potential analysis confirmed that the surface electronegativity of the PAs intensified with increasing DP. These characteristics provide a structural and physicochemical basis for the interactions between PAs and digestive enzymes, which were further investigated using inhibition assays, kinetic analysis, fluorescence spectroscopy, turbidity measurements, and SEM observation.

### 3.3 Inhibition of digestive enzymes in vitro

Based on the established structural characteristics related to DP, we further examined whether these properties contributed to the enhanced inhibitory effects against Pep, Try, Lip, and Amy. All PA fractions exhibited dose-dependent inhibitory behavior (Fig. 2A), and their inhibitory potency was quantified in terms of IC₅₀ values (Fig. 2B). The IC₅₀ ranges of F_1_-F_8_ against Pep, Try, Lip, and Amy were 11.05–0.05, 0.62–0.01, 0.74–0.02, and 0.62–0.08 mg/mL, respectively. The tested enzymes displayed differential susceptibility to PA inhibition, following the order Try ≈ Lip ≈ Amy > Pep. Notably, IC₅₀ values decreased progressively with increasing DP, indicating a positive correlation between DP and inhibitory capacity. This phenomenon implies that highly polymerized PAs possess a stronger enzyme-inhibiting potential.

**Fig. 2:**
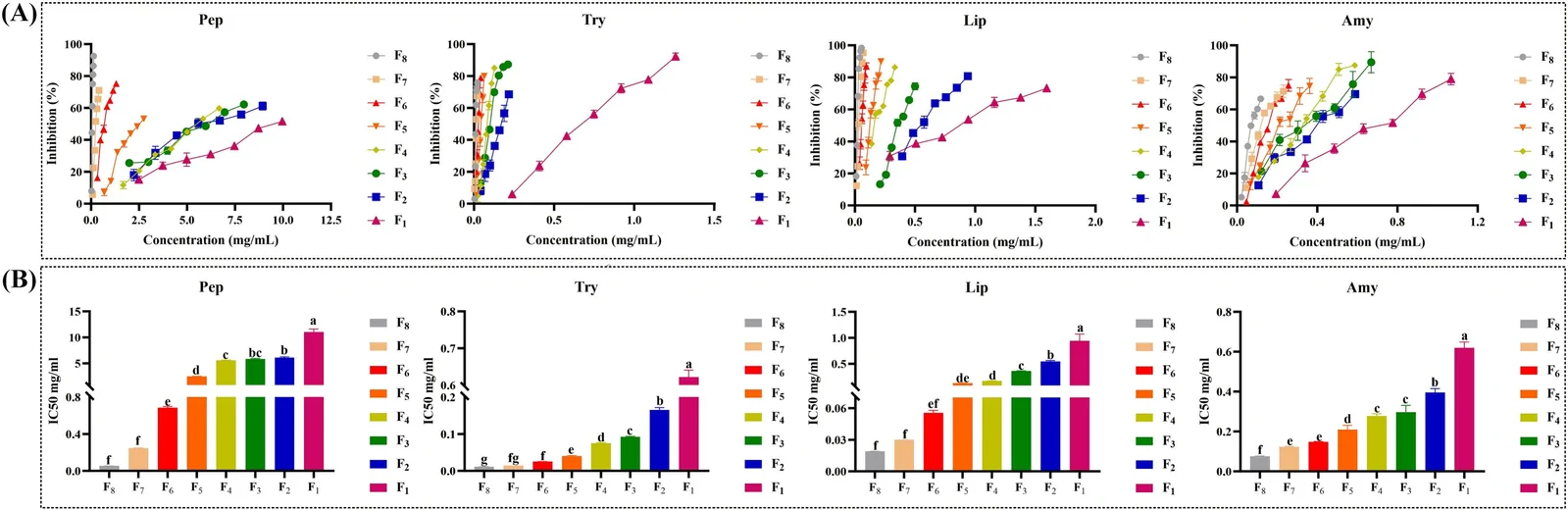
(A) Inhibitory effects of various concentrations of proanthocyanidins with different degrees of polymerization (F_1_-F_8_) on pepsin (Pep), trypsin (Try), lipase (Lip), and *α*-amylase (Amy). (B) Half-maximal inhibitory concentration (IC₅₀) values of proanthocyanidins with different degrees of polymerization (F_1_-F_8_) against pepsin (Pep), trypsin (Try), lipase (Lip), and *α*-amylase (Amy). Different letters indicate significant differences (*p* < 0.05).

Previous molecular simulation studies have demonstrated that low-DP PAs preferentially enter internal enzyme cavities, whereas high-DP PAs tend to bind to enzyme exterior surfaces (Vazquez-Flores et al., 2018; Zhou et al., 2018). Although such binding-site discrepancies partially differentiate the interaction modes of PA polymers, this structural variation alone cannot fully rationalize the superior inhibitory efficacy of high-DP fractions, indicating the participation of additional intermolecular mechanisms. Multi-site synergistic binding has been validated to strengthen the molecular binding affinity and inhibitory efficiency (Bienstein et al., 2022; Osmaniye et al., 2019; Wang et al., 2016). Accordingly, we hypothesized that multi-site synergistic binding predominantly governs the DP-related inhibitory improvement of PAs. High-DP PAs contain larger molecular skeletons and abundant binding sites, which facilitate extensive synergistic interactions with enzyme surfaces, thereby conferring a higher binding affinity. Coupled with the enhanced surface electronegativity verified by zeta potential analysis, the intensified electrostatic attraction further facilitated the PA-enzyme combination. Therefore, further kinetic and spectroscopic analyses were performed to validate this synergistic binding mechanism and clarify the structure-inhibition relationship of PA molecules.

### 3.4 Kinetic type of inhibition

#### 3.4.1 Reversibility

Plots of enzyme velocity (*ν*) versus enzyme concentration (Pep, Try, Lip, and Amy) at different concentrations of F_1_-F_8_ were constructed to evaluate the reversibility of inhibition against Pep, Try, Lip, and Amy (Fig. S3-S6). Upon the addition of F_1_-F_8_, all straight lines passed through the origin with decreasing slopes, suggesting that the inhibition mediated by F_1_-F_8_ was a reversible process and involved noncovalent intermolecular interactions between F_1_-F_8_ and Pep, Try, Lip, and Amy. If the inhibition is irreversible, the enzyme is inactivated through the covalent binding between the inhibitor and the enzyme, leading to the formation of a stable complex. In such cases, the plots of *ν* versus enzyme concentration do not pass through the origin (Xu et al., 2015). In contrast, the linear and origin-passing characteristics observed in the present study further confirmed the reversible inhibitory nature of all tested PA fractions.

#### 3.4.2 Type of digestive enzyme inhibition

Lineweaver-Burk plots were used to examine the inhibitory effects of PA fractions (F_1_-F_8_) on the four digestive enzymes. As depicted in Fig. 3, the data demonstrated a strong correlation (R^2^ > 0.98) with no evidence of substrate inhibition. This suggests that the kinetic data are valid and reliable for characterizing the reversible inhibition behavior of all PA fractions. Most PA-enzyme combinations (PA-Pep, PA-Lip, and PA-Amy) exhibited intersection points in the first quadrant, which did not conform to standard categories, such as competitive, non-competitive, or uncompetitive inhibition. This indicates a complex and non-standard inhibition mechanism. The nonlinear dependence of the curve slopes on PA concentration further suggested that PAs likely bind to non-active-site regions, inducing conformational rearrangements, which is indicative of allosteric regulation.

**Fig. 3:**
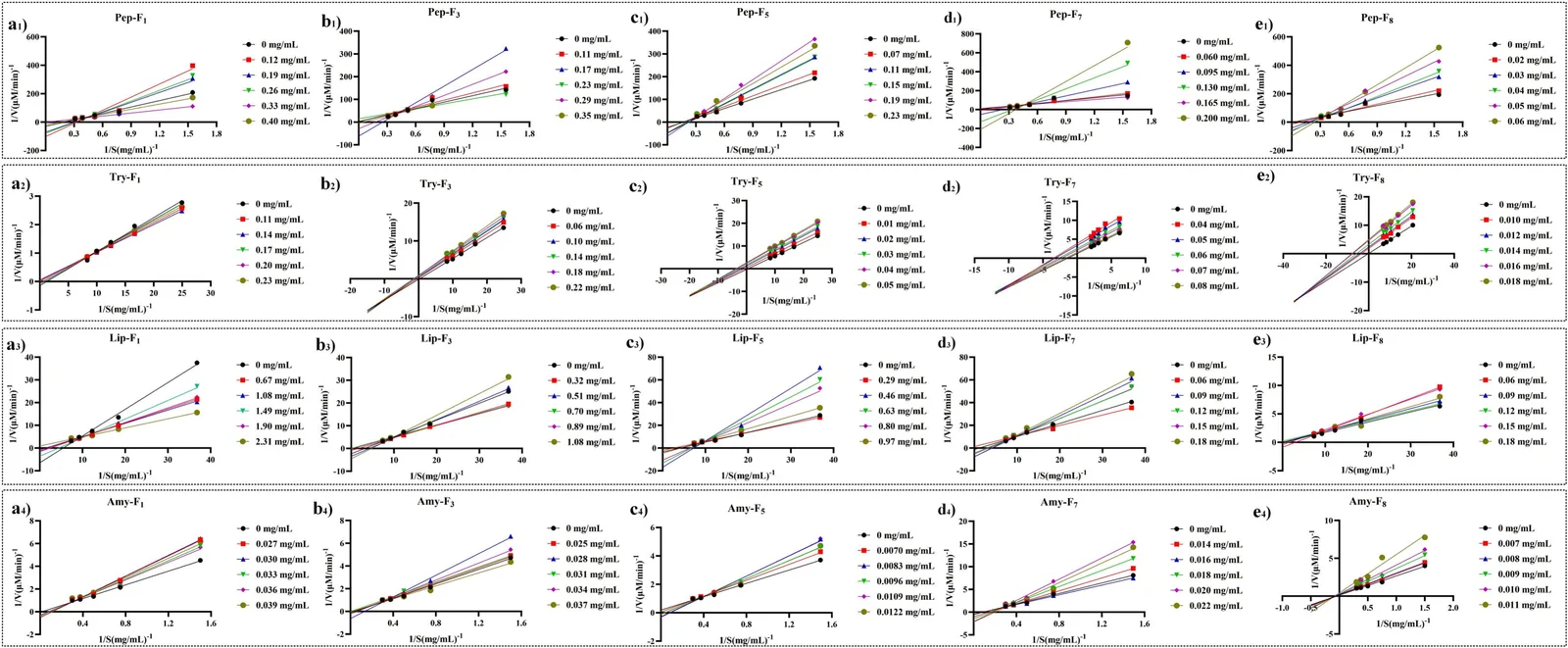
The Lineweaver-Burk plots for the inhibition of pepsin (Pep), trypsin (Try), lipase (Lip), and *α*-amylase (Amy) by proanthocyanidin fractions with different degrees of polymerization (F_1_-F_8_) at various concentrations.

Hill coefficients (nH) were used to assess allosteric cooperativity. If nH is not equal to 1 (nH > 1 means positive cooperativity; nH < 1 means negative cooperativity), it demonstrates cooperative and allosteric behavior (Stefan & Le Novère, 2013). Table S2 reveals that most nH values ranged between 1.00 and 1.90, indicating that the majority of PA fractions exhibited positive cooperativity toward the four enzymes. A few systems, such as F_7_-Try and F_8_-Lip, exhibited negative cooperativity (nH < 1.0) at certain concentrations of the substrates. This transition between positive and negative cooperativity is attributed to the differential occupancy of high- and low-affinity binding sites and their associated conformational states (Grant et al., 1996). The nH values reflect multi-site binding synergism (Newell & Schachman, 1990; Weiss, 1997). The concentration-independent fluctuations in nH observed in this study further suggest that PAs with varying DP may trigger intricate allosteric transitions via multi-site interactions.

The inhibitory behavior of the compounds toward Try was examined independently. As illustrated in Fig. 4, for F_3_-F_8_, the lines at various inhibitor concentrations intersect in the third quadrant. Both Km and Vmax decreased as the inhibitor concentration increased, indicating mixed-type inhibition with uncompetitive characteristics. Uncompetitive characteristics imply that the inhibitor can bind to both the free enzyme and the enzyme-substrate (ES) complex, forming enzyme-substrate-inhibitor (ESI) complexes (Sharma, 2012; Silva et al., 2023). These data demonstrate that these PAs can associate with both free enzymes and enzyme-substrate complexes to form ESI complexes, thereby reducing catalytic efficiency and product release. In contrast, F_1_ exhibited a distinct inhibitory pattern similar to that observed for the other three enzymes.

**Fig. 4:**
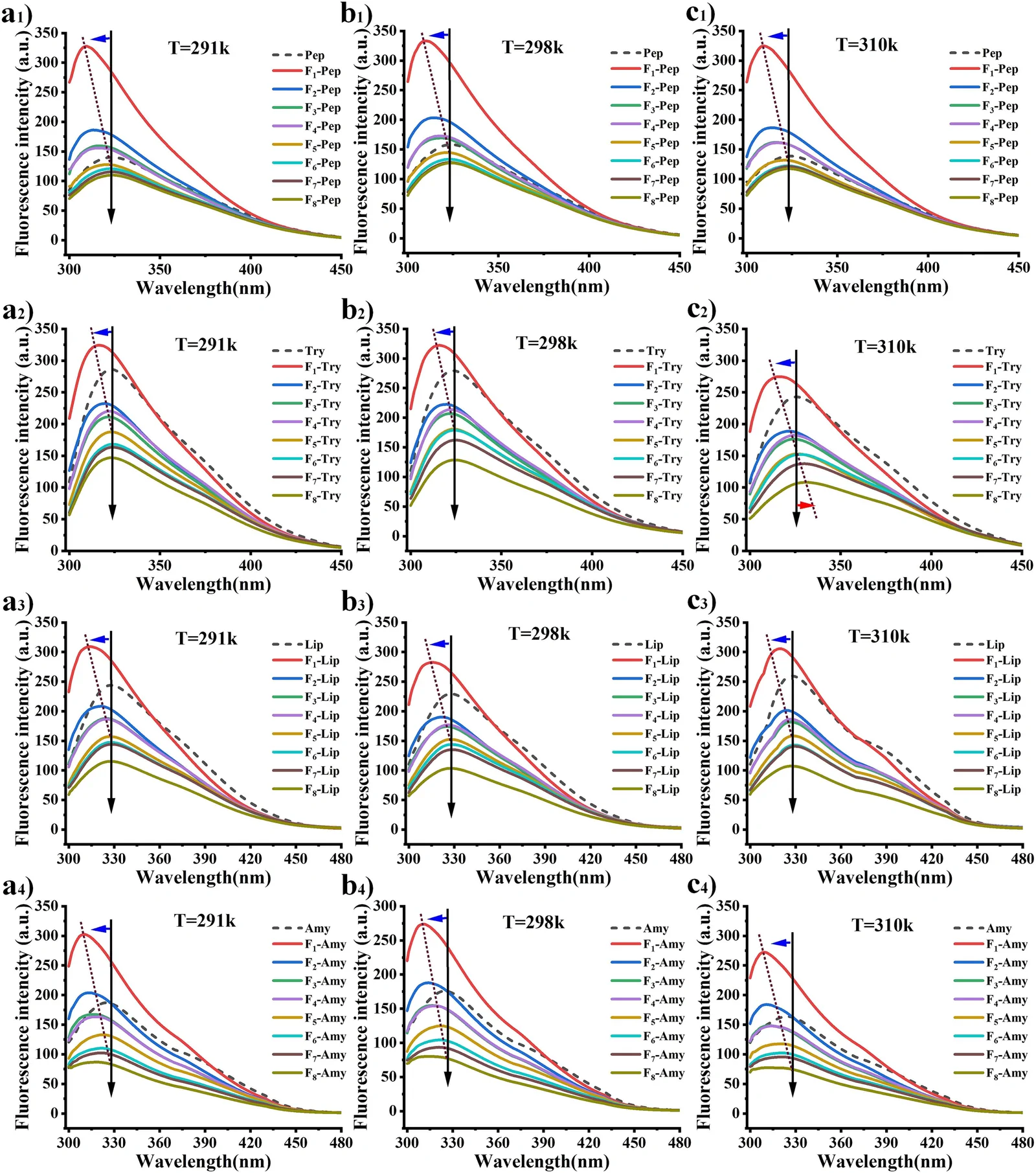
Fluorescence emission spectra of pepsin (Pep), trypsin (Try), lipase (Lip), and *α*-amylase (Amy) in the presence of proanthocyanidins with different degrees of polymerization (F_1_-F_8_) at the same concentration at 291, 298, and 310 K.

Distinct Hill coefficients and atypical inhibition profiles reveal complex interaction modes between diverse PA fractions and digestive enzymes. Multi-site allosteric binding and ternary ESI complex formation could induce conformational changes in enzymes and trigger the aggregation of PA-enzyme complexes under specific conditions. Turbidity detection, spectroscopic analysis, and morphological observation further verified the inhibition mechanism associated with aggregate formation.

### 3.5 Turbidity analysis

Turbidity measurement was utilized to characterize the aggregation behavior between PA fractions and four digestive enzymes by monitoring particle light-scattering intensity. This method is widely employed to evaluate polyphenol-protein aggregation, as larger and more stable assemblies typically result in higher absorbance values (Leppä et al., 2020; Ropiak et al., 2017). Consequently, variations in turbidity were used to reflect the extent of complexation and the relative abundance of aggregates in PA-enzyme mixtures. Turbidity across PA-to-enzyme mass ratios is summarized in Table 2. At a low mass ratio (1:3), turbidity increased progressively with rising DP. This trend was attributed to the larger molecular dimensions of high-DP PAs, which facilitate the formation of larger assemblies at low PA concentrations. These observations are consistent with previous reports that long-chain polyphenols exhibit a stronger protein-aggregating propensity (Leppä et al., 2020; Ropiak et al., 2017). Higher PA-to-enzyme ratios (1:1 and 3:1) significantly elevated absorbance, indicating extensive aggregate formation under PA-rich conditions. However, no strict linear correlation between turbidity and DP was observed. Similar non-monotonic turbidity patterns have been reported in other polyphenol-protein systems, wherein excess polyphenols induce microstructural remodeling rather than continuous aggregate growth (Brás et al., 2010). Such microstructural remodeling accounts for the nonlinear relationship between turbidity and DP, implying that the extent of aggregation between PAs and digestive enzymes does not follow a simple stoichiometric relationship but likely involves multi-site cooperative and diversified interaction modes. This observation is consistent with multi-site cooperativity identified in kinetic analyses, suggesting that multi-site cooperative interactions govern the macroscopic assembly of PA-enzyme complexes.

**Table 2:** Turbidity of pepsin (Pep), trypsin (Try), lipase (Lip), and *α*-amylase (Amy) treated with proanthocyanidins of different degrees of polymerization (F_1_-F_8_) at different mass ratios.

Fraction	Fx:Pep			Fx:Try			Fx:Lip			Fx:Amy		
	3:1	1:1	1:3	3:1	1:1	1:3	3:1	1:1	1:3	3:1	1:1	1:3
F_1_	0.063 ± 0.00^d^	0.048 ± 0.00^f^	0.040 ± 0.00^d^	0.056 ± 0.00^e^	0.043 ± 0.00^d^	0.038 ± 0.00^f^	0.072 ± 0.00^f^	0.048 ± 0.00^e^	0.040 ± 0.00^g^	0.075 ± 0.00^e^	0.048 ± 0.00^f^	0.039 ± 0.00^d^
F_2_	0.085 ± 0.00^d^	0.052 ± 0.00^ef^	0.042 ± 0.00^d^	0.061 ± 0.00^d^	0.044 ± 0.00^d^	0.038 ± 0.00^ef^	0.128 ± 0.00^e^	0.057 ± 0.00^e^	0.042 ± 0.00^f^	0.093 ± 0.00^de^	0.058 ± 0.00^ef^	0.043 ± 0.00^cd^
F_3_	0.122 ± 0.00^cd^	0.062 ± 0.00^ef^	0.044 ± 0.00^d^	0.095 ± 0.00^c^	0.055 ± 0.00^c^	0.041 ± 0.00^de^	0.177 ± 0.00^d^	0.091 ± 0.00^d^	0.046 ± 0.00^e^	0.157 ± 0.00^b^	0.074 ± 0.00^de^	0.047 ± 0.00^cd^
F_4_	0.173 ± 0.00^c^	0.086 ± 0.00^d^	0.051 ± 0.00^cd^	0.091 ± 0.00^c^	0.056 ± 0.00^c^	0.041 ± 0.00^cd^	0.137 ± 0.00^e^	0.083 ± 0.00^d^	0.048 ± 0.00^d^	0.126 ± 0.00^d^	0.067 ± 0.00^cde^	0.047 ± 0.00^cd^
F_5_	0.097 ± 0.01^d^	0.070 ± 0.00^de^	0.062 ± 0.00^c^	0.091 ± 0.00^c^	0.058 ± 0.00^c^	0.044 ± 0.00^c^	0.198 ± 0.00^d^	0.112 ± 0.00^c^	0.066 ± 0.00^c^	0.101 ± 0.01^c^	0.064 ± 0.00^cd^	0.050 ± 0.00^c^
F_6_	0.343 ± 0.03^b^	0.125 ± 0.03^c^	0.085 ± 0.00^b^	0.100 ± 0.00^b^	0.065 ± 0.00^b^	0.051 ± 0.00^b^	0.357 ± 0.01^c^	0.124 ± 0.00^c^	0.080 ± 0.00^b^	0.129 ± 0.00^c^	0.080 ± 0.00^c^	0.061 ± 0.00^b^
F_7_	0.349 ± 0.04^b^	0.205 ± 0.01^b^	0.089 ± 0.00^b^	0.103 ± 0.00^b^	0.067 ± 0.00^b^	0.054 ± 0.00^a^	0.387 ± 0.01^b^	0.141 ± 0.00^b^	0.080 ± 0.00^b^	0.141 ± 0.00^bc^	0.101 ± 0.00^b^	0.065 ± 0.00^b^
F_8_	1.391 ± 0.07^a^	0.915 ± 0.01^a^	0.276 ± 0.02^a^	0.177 ± 0.01^a^	0.091 ± 0.01^a^	0.056 ± 0.00^a^	0.782 ± 0.04^a^	0.258 ± 0.02^a^	0.105 ± 0.00^a^	0.556 ± 0.03^a^	0.207 ± 0.02^a^	0.158 ± 0.01^a^

Abbreviations: Different lowercase letters indicate significant differences (*p* < 0.05).

### 3.6 Mechanisms underlying interactions between PAs with different DP and digestive enzymes

By integrating the structural features, inhibition kinetics, and turbidity characteristics, it is evident that the functional properties of PAs are intricately linked to their DP. Generally, PAs with a high DP exhibit increased surface electronegativity and a reduced IC₅₀ value. Furthermore, PAs with varying DP may exhibit reversible inhibitory effects through multiple-site cooperative interactions.

However, the molecular mechanisms underlying these macroscopic phenomena remain unclear. Three critical issues require further exploration: (i) the effects of PAs with varying DP on the tryptophan (Trp) microenvironment and binding equilibrium of enzymes; (ii) the conformational changes of enzymes induced by PA binding; and (iii) the steric hindrance effects of molecular alterations on aggregate morphology and substrate accessibility. Therefore, fluorescence spectroscopy, circular dichroism, FTIR spectroscopy, and SEM were used to clarify the intrinsic structure-activity relationship of PAs.

#### 3.6.1 Fluorescence spectroscopy analysis

Endogenous protein fluorescence predominantly arises from Trp residues, and Trp fluorescence is highly sensitive to alterations in the microenvironment and protein conformation (Dos Santos Rodrigues et al., 2023). Fluorescence spectroscopy was employed to examine Trp microenvironmental changes during the interaction between the four digestive enzymes and PA fractions with varying DP. Fig. 4 presents the fluorescence spectra of the enzymes following incubation with fractions F_1_-F_8_ for 30 min at an excitation wavelength of 280 nm. The intrinsic *λ*max values for Pep, Try, Lip, and Amy were 335, 335, 336, and 341 nm, respectively. Upon treatment with PAs of different DP, the *λ*max values of all enzymes exhibited varying degrees of shift. Trp residues typically produce a characteristic emission peak near 350 nm, where blue shifts indicate decreased polarity and increased hydrophobicity, whereas red shifts suggest a more polar microenvironment (Ghisaidoobe & Chung, 2014; Wang et al., 2023; Zhang et al., 2022). Treatment with PAs of varying DP induced distinct *λ*max shifts in all enzymes, indicating that PAs with varying DP modulated the polarity of the Trp microenvironment.

Further comparative analysis indicated that the *λ*max values of various digestive enzymes for PAs with varying DP exhibited discontinuous responses and distinct thresholds. The blue shifts in *λ*max for Pep and Lip gradually diminished below an mDP of 4 and remained stable at mDP exceeding 4, suggesting a threshold near 4. In contrast, Try and Amy demonstrated higher thresholds of approximately 5.5 and 12.5, respectively. The PA fractions also induced notable DP-related variations in fluorescence intensity. For Pep, the fluorescence intensity increased at mDP < 4 and was progressively quenched at mDP exceeding 4. Similar quenching thresholds were observed for Try, Lip, and Amy at mDP values of 2, 3, and 2.5, respectively. The inconsistent thresholds between the wavelength shift and intensity variation suggest that Trp polarity alteration and fluorescence quenching arise from different PA-induced structural perturbations.

The combined variations in *λ*max and fluorescence intensity indicate that DP-related transitions occur in the interaction patterns between PAs and digestive enzymes. Low-DP PAs elicited blue shifts and moderate fluorescence enhancement, implying increased hydrophobicity around Trp residues without substantial conformational perturbation of the enzyme. Beyond the critical fluorescence quenching DP threshold, pronounced fluorescence quenching occurred, accompanied by attenuated or reversed *λ*max shifts, suggesting that high-DP PAs triggered extensive conformational rearrangement and intermolecular aggregation.

Concentration-dependent spectral analyses further corroborated these findings (Fig. S7-S10). For PAs with an mDP less than 4, increased PA concentrations resulted in intensified blue shifts for all enzymes. Conversely, fractions with mDP greater than 4 elicited enzyme-specific responses: Pep and Amy consistently exhibited blue shifts, whereas Try and Lip demonstrated concentration-dependent red shifts. These distinct spectral behaviors were attributed to multi-site interactions, which induced varying degrees of enzyme conformational changes at elevated PA concentrations. When the degree of aggregation exceeds the fluorescence quenching threshold, the extent of quenching increases with concentration, enabling a more effective analysis of fluorescence quenching-related parameters.

The Stern-Volmer equation was employed to examine the fluorescence quenching mechanisms of the four digestive enzymes induced by PA fractions. The Stern-Volmer plots exhibited good linearity across different temperatures, suggesting either static or dynamic quenching behavior (Fossum et al., 2023). All the quenching parameters are listed in Table 3. PA fractions exceeding specific DP thresholds resulted in reduced fluorescence intensity, facilitating the valid calculations of Ksv and Kq. Conversely, subthreshold PAs increased fluorescence intensity, merely reflecting microenvironmental changes without genuine quenching (Fossum et al., 2023); therefore, these groups were excluded from further mechanistic analysis. For PAs above the thresholds, the Stern-Volmer quenching constant (Ksv) ranged from 0.07 × 10^6^ to 1.36 × 10^9^ L·mol^−1^, while the quenching rate constant (Kq) varied from 0.07 × 10^14^ to 1.37 × 10^16^ L·mol^−1^·s^−1^. These Kq values consistently exceeded the maximum collisional quenching constant typical of dynamic quenching (2.0 × 10^10^ L·mol^−1^·s^−1^), and Ksv decreased with increasing temperature, both of which are indicative of a static quenching mechanism (Wang et al., 2023). These observations confirm that static quenching predominates in the interaction process, indicating the spontaneous formation of a non-fluorescent PA-enzyme ground state complex.

**Table 3:** Fluorescence quenching parameters and thermodynamic parameters of pepsin (Pep), trypsin (Try), lipase (Lip), and *α*-amylase (Amy) treated with proanthocyanidins of different degrees of polymerization (F_1_-F_8_) at varying concentrations at 291, 298, and 310 K.

	T (K)	K_sv_ (10^6^ L/moL)	K_q_ (10^14^ L/moL)	R^2^	K_a_ (10^4^ L/moL)	n	∆H (kJ/moL)	∆S (J/moL·K^−1^)	∆G (kJ/moL)
F_1_-Pep	291	−0.31 ± 0.00^c^	−0.03 ± 0.00^d^	0.90	–	–	–	–	–
	298	−0.39 ± 0.01^c^	−0.39 ± 0.01^d^	0.89	–	–			–
	310	−0.03 ± 0.00^c^	−0.03 ± 0.00^d^	0.90	–	–			–
F_2_-Pep	291	−0.80 ± 0.01^c^	−0.08 ± 0.00^d^	0.92	–	–	–	–	–
	298	−0.83 ± 0.01^c^	−0.83 ± 0.01^d^	0.94	–	–			–
	310	−0.08 ± 0.01^c^	−0.08 ± 0.01^d^	0.92	–	–			–
F_3_-Pep	291	−0.78 ± 0.07^c^	−0.08 ± 0.01^d^	0.94	–	–	–	–	–
	298	−0.76 ± 0.05^c^	−0.76 ± 0.05^d^	0.93	–	–			–
	310	−0.06 ± 0.00^c^	−0.06 ± 0.00^d^	0.88	–	–			–
F_4_-Pep	291	−0.95 ± 0.07^c^	−0.10 ± 0.01^d^	0.98	–	–	–	–	–
	298	−0.83 ± 0.06^c^	−0.83 ± 0.06^d^	0.96	–	–			–
	310	−0.08 ± 0.00^c^	−0.08 ± 0.00^d^	0.97	–	–			–
F_5_-Pep	291	0.68 ± 0.11^c^	0.07 ± 0.01^d^	0.91	0.0453	0.49 ± 0.04^g^	0.0002	0.02968	−8.6375
	298	1.39 ± 0.16^c^	1.39 ± 0.16^d^	0.99	3.2470	0.76 ± 0.11^ef^			−8.8453
	310	0.07 ± 0.01^c^	0.07 ± 0.01^d^	0.98	1.1902	0.72 ± 0.09^f^			−9.2017
F_6_-Pep	291	3.30 ± 0.67^c^	0.33 ± 0.07^d^	0.98	107.3568	0.91 ± 0.09^cde^	0.0006	0.03664	−10.6623
	298	4.73 ± 0.37^c^	4.73 ± 0.37^c^	0.98	400.5091	0.99 ± 0.04^bcd^			−10.9188
	310	0.33 ± 0.00^c^	0.33 ± 0.00^d^	0.96	1827.6426	1.10 ± 0.10^ab^			−11.3591
F_7_-Pep	291	74.69 ± 10.11^b^	7.47 ± 1.01^b^	1.00	619.6220	0.99 ± 0.07^bcd^	0.0006	0.03781	−11.0024
	298	8.24 ± 0.85^c^	8.24 ± 0.85^b^	0.99	2360.4730	1.07 ± 0.04^abc^			−11.2671
	310	7.13 ± 0.33^c^	7.13 ± 0.33^bc^	0.99	15,836.3370	1.20 ± 0.04^a^			−11.7214
F_8_-Pep	291	116.56 ± 60.29^a^	11.66 ± 6.03^a^	0.95	79.2795	0.82 ± 0.21^def^	0.0011	0.04552	−13.2448
	298	12.71 ± 3.63^c^	12.71 ± 3.63^a^	0.98	1057.2072	0.99 ± 0.12^bcd^			−13.5634
	310	11.24 ± 1.31^c^	11.24 ± 1.31^a^	0.98	3549.8598	1.07 ± 0.04^abc^			−14.1107
F_1_-Try	291	−2.91 ± 0.43^d^	−0.29 ± 0.04^j^	0.99	–	–	–	–	–
	298	−0.31 ± 0.10^d^	−0.31 ± 0.10^j^	0.97	–	–			–
	310	−0.39 ± 0.03^d^	−0.39 ± 0.03^j^	0.97	–	–			–
F_2_-Try	291	14.91 ± 1.41^d^	1.49 ± 0.14^j^	0.9	0.0688	0.62 ± 0.07^m^	0.0035	0.05217	−15.1787
	298	1.67 ± 0.18^d^	1.67 ± 0.18^j^	0.97	0.1073	0.66 ± 0.02^lm^			−15.5439
	310	2.01 ± 0.11^d^	2.01 ± 0.11^j^	0.92	0.1987	0.66 ± 0.06^lm^			−16.1734
F_3_-Try	291	30.43 ± 2.26^d^	3.04 ± 0.23^ij^	0.99	0.3327	0.72 ± 0.04^kl^	0.0010	0.03648	−10.6145
	298	3.21 ± 0.29^d^	3.21 ± 0.29^ij^	1.00	0.7460	0.78 ± 0.03^jk^			−10.8698
	310	4.15 ± 0.34^d^	4.15 ± 0.34^hij^	0.98	4.0499	0.85 ± 0.06^ij^			−11.3085
F_4_-Try	291	25.80 ± 2.47^d^	2.58 ± 0.25^j^	0.99	0.1381	0.65 ± 0.05^lm^	0.0003	0.03082	−8.9692
	298	4.24 ± 0.34^d^	4.24 ± 0.34^hij^	1.00	6.0847	0.95 ± 0.02^h^			−9.1849
	310	4.58 ± 0.19^d^	4.58 ± 0.19^hij^	0.99	5.5113	0.90 ± 0.03^hi^			−9.5551
F_5_-Try	291	107.43 ± 2.58^c^	10.74 ± 0.26^ghi^	0.99	18.9661	0.97 ± 0.02^gh^	−0.0004	0.02276	−6.6245
	298	12.13 ± 0.67^d^	12.13 ± 0.67^fg^	1.00	50.1781	1.05 ± 0.01^fg^			−6.7839
	310	11.62 ± 0.25^d^	11.62 ± 0.25^fgh^	1.00	19.0117	0.95 ± 0.02^h^			−7.0566
F_6_-Try	291	197.84 ± 6.05^c^	19.78 ± 0.61^e^	0.99	71.0791	1.03 ± 0.01^g^	−0.0001	0.02678	−7.7919
	298	18.43 ± 0.69^d^	18.43 ± 0.69^ef^	0.99	351.3645	1.18 ± 0.03^de^			−7.9793
	310	17.27 ± 0.72^d^	17.27 ± 0.72^efg^	0.98	118.3959	1.05 ± 0.04^fg^			−8.3006
F_7_-Try	291	310.21 ± 21.56^b^	31.02 ± 2.16^d^	0.99	356.9846	1.13 ± 0.06^ef^	−0.0005	0.02028	−5.9024
	298	33.10 ± 2.48^d^	33.10 ± 2.48^d^	0.99	1202.7138	1.22 ± 0.03^cd^			−6.0444
	310	27.48 ± 1.35^d^	27.48 ± 1.35^d^	1.00	207.7768	1.05 ± 0.04^fg^			−6.2873
F_8_-Try	291	847.13 ± 150.53^a^	84.71 ± 15.05^c^	0.99	12,424.9150	1.32 ± 0.12^b^	−0.0003	0.02231	−6.4914
	298	127.61 ± 12.53^c^	127.61 ± 12.53^b^	0.99	179,291.0800	1.50 ± 0.05^a^			−6.6475
	310	98.25 ± 5.06^c^	98.25 ± 5.06^a^	0.98	13,722.1200	1.26 ± 0.05^bc^			−6.9149
F_1_-Lip	291	−5.72 ± 0.61^g^	−0.57 ± 0.06^f^	0.99	–	–	–	–	–
	298	−0.63 ± 0.02^fg^	−0.63 ± 0.02^f^	0.96	–	–			–
	310	−0.42 ± 0.02^fg^	−0.42 ± 0.02^f^	0.95	–	–			–
F_2_-Lip	291	3.61 ± 2.41^fg^	0.36 ± 0.24^f^	0.84	2.8983	0.19 ± 0.10^j^	0.0001	0.0286	−8.3176
	298	0.44 ± 0.02^fg^	0.44 ± 0.02^f^	0.65	0.0010	0.22 ± 0.02^j^			−8.5177
	310	2.20 ± 0.94^fg^	2.20 ± 0.94^f^	0.67	0.0007	0.68 ± 0.22^i^			−8.8607
F_3_-Lip	291	29.67 ± 3.42^defg^	2.97 ± 0.34^f^	0.98	1389.9124	0.75 ± 0.03^i^	0.0002	0.0304	−8.8578
	298	2.82 ± 0.08^fg^	2.82 ± 0.08^f^	0.99	2.9557	0.69 ± 0.02^i^			−9.0709
	310	5.65 ± 1.08^efg^	5.65 ± 1.08^fg^	0.99	7.5616	1.05 ± 0.10^gh^			−9.4362
F_4_-Lip	291	31.88 ± 3.66^defg^	3.19 ± 0.37^f^	0.99	3909.2595	0.71 ± 0.02^i^	0.0002	0.0309	−8.9852
	298	3.41 ± 0.13^fg^	3.41 ± 0.13^f^	1.00	14.3620	0.79 ± 0.06^i^			−9.2014
	310	6.67 ± 1.11^efg^	6.67 ± 1.11^fg^	1.00	4.6438	1.12 ± 0.09^efgh^			−9.5719
F_5_-Lip	291	112.41 ± 9.91^def^	11.24 ± 0.99^efg^	1.00	20,753.4440	0.97 ± 0.01^h^	0.0005	0.0359	−10.4313
	298	12.42 ± 0.32^efg^	12.42 ± 0.32^efg^	0.99	4920.8643	1.09 ± 0.02^fgh^			−10.6822
	310	16.05 ± 2.44^efg^	16.05 ± 2.44^efg^	1.00	740.3430	1.16 ± 0.08^efg^			−11.1124
F_6_-Lip	291	224.51 ± 16.74^c^	22.45 ± 1.67^def^	1.00	76,364.0580	1.20 ± 0.01^defg^	0.0014	0.0563	−16.3736
	298	21.85 ± 0.49^efg^	21.85 ± 0.49d^ef^	0.99	107,955.1900	1.25 ± 0.02^bcde^			−16.7675
	310	26.84 ± 3.09^efg^	26.84 ± 3.09^cde^	0.99	45,796.4290	1.21 ± 0.07^cdef^			−17.4428
F_7_-Lip	291	391.77 ± 30.74^b^	39.18 ± 3.07^c^	1.00	640,262.7900	1.38 ± 0.00^b^	−0.0001	0.0265	−7.7158
	298	33.01 ± 1.56^defg^	33.01 ± 1.56^cd^	0.99	168,256.1700	1.25 ± 0.00^bcde^			−7.9014
	310	40.98 ± 5.65^defg^	40.98 ± 5.65^c^	0.99	1,564,732.3000	1.32 ± 0.08^bcd^			−8.2196
F_8_-Lip	291	1358.07 ± 278.31^a^	135.81 ± 27.83^a^	1.00	3,346,749.7000	1.54 ± 0.13^a^	−0.0004	0.0184	−5.3615
	298	117.61 ± 22.35^de^	117.61 ± 22.35^b^	0.99	3,740,922.8000	1.35 ± 0.11b^c^			−5.4904
	310	137.42 ± 24.64^cd^	137.42 ± 24.64^a^	0.99	96,207,559.0000	1.34 ± 0.05^bcd^			−5.7115
F_1_-Amy	291	−5.96 ± 0.32^e^	−0.60 ± 0.03^i^	0.97	–	–	–	–	–
	298	−0.62 ± 0.01^e^	−0.62 ± 0.01^i^	0.95	–	–			–
	310	−0.54 ± 0.02^e^	−0.54 ± 0.02^i^	0.96	–	–			–
F_2_-Amy	291	−6.51 ± 0.40^e^	−0.65 ± 0.04^i^	0.96	–	–	–	–	–
	298	−0.67 ± 0.05^e^	−0.67 ± 0.05^i^	0.91	–	–			–
	310	−0.65 ± 0.04^e^	−0.65 ± 0.04^i^	0.89	–	–			–
F_3_-Amy	291	−2.95 ± 0.90^e^	−0.29 ± 0.09^i^	0.86	0.7474	−0.37 ± 0.15^de^	−0.0003	0.0303	−8.8207
	298	−0.38 ± 0.08^e^	−0.38 ± 0.08^i^	0.89	0.0002	−0.44 ± 0.10^ef^			−9.0328
	310	−0.28 ± 0.04^e^	−0.28 ± 0.04^i^	0.78	0.0004	−0.59 ± 0.03^f^			−9.3966
F_4_-Amy	291	−2.80 ± 1.46^e^	−0.28 ± 0.15^i^	0.75	0.0000	−0.22 ± 0.12^d^	−0.0004	0.0303	−8.8295
	298	−0.44 ± 0.07^e^	−0.44 ± 0.07^i^	0.95	0.0000	−0.32 ± 0.04^de^			−9.0419
	310	−0.46 ± 0.13^e^	−0.46 ± 0.13^i^	0.88	0.0006	−0.57 ± 0.25^f^			−9.406
F_5_-Amy	291	54.28 ± 7.52^d^	5.43 ± 0.75^ghi^	0.99	0.0043	0.67 ± 0.06^ab^	−0.0006	0.0226	−6.5681
	298	4.89 ± 0.33^e^	4.89 ± 0.33^hi^	0.97	0.0040	0.60 ± 0.03^bc^			−6.7261
	310	3.44 ± 0.51^e^	3.44 ± 0.51^hi^	0.94	0.0086	0.48 ± 0.06^c^			−6.9969
F_6_-Amy	291	125.19 ± 13.14^c^	12.52 ± 1.31^ef^	0.96	0.0044	0.72 ± 0.04^ab^	−0.0008	0.0190	−5.5215
	298	10.76 ± 0.81^de^	10.76 ± 0.81^fg^	0.95	0.0079	0.64 ± 0.04^bc^			−5.6543
	310	8.56 ± 0.83^de^	8.56 ± 0.83^fgh^	0.95	0.0149	0.60 ± 0.04^a^			−5.8819
F_7_-Amy	291	242.03 ± 21.69^b^	24.20 ± 2.17^c^	0.99	0.0083	0.83 ± 0.03^a^	−0.0005	0.0208	−6.038
	298	19.41 ± 3.23^de^	19.41 ± 3.23^cd^	0.98	0.0304	0.68 ± 0.08^ab^			−6.1832
	310	16.70 ± 1.51^de^	16.70 ± 1.51^de^	0.97	0.0226	0.73 ± 0.03^ab^			−6.4322
F_8_-Amy	291	409.41 ± 119.00^a^	40.94 ± 11.90^a^	0.88	0.0021	0.68 ± 0.10^ab^	−0.0007	0.0197	−5.7285
	298	34.95 ± 7.58^de^	34.95 ± 7.58^b^	0.97	0.0077	0.59 ± 0.10^bc^			−5.8663
	310	31.48 ± 5.48^de^	31.48 ± 5.48^b^	0.89	0.0283	0.61 ± 0.05^bc^			−6.1025

Abbreviations: For values within the same column, significance analysis indicated by different letters is limited to comparisons among F_1_-F_8_ fractions for the same digestive enzyme. Different lowercase letters represent significant differences (*p* < 0.05).

The binding constants (Ka) and number of binding sites (n) were determined using the modified Stern-Volmer equation (Cai et al., 2015). The relevant fitting data are shown in Fig. S15-S18 and Table 3. The Ka value decreased with increasing temperature, consistent with the characteristics of static quenching (Li et al., 2016), thereby corroborating the static quenching properties of different DP of PAs and enzymes. When the mDP of PAs exceeded the polymerization threshold responsible for fluorescence quenching, the K_a_ values for Pep, Try, Lip, and Amy ranged from 0.05 × 10^4^ to 1.58 × 10^8^, 0.07 × 10^4^ to 1.79 × 10^9^, 0.01 × 10^4^ to 9.62 × 10^11^, and 1.00 to 47.40 L·mol^−1^, respectively. For concentrations exceeding the threshold, significant variations in Ka values were observed among the different enzyme systems. According to the universal binding strength criteria (weak binding: Ka < 10^4^ L·mol^−1^; moderate binding: 10^4^ ≤ Ka < 10^6^ L·mol^−1^; strong binding: Ka ≥ 10^6^ L·mol^−1^) (Cai et al., 2015; Li et al., 2016; Prigent et al., 2009), Pep, Try, and Lip exhibited moderate-to-strong binding, while Amy showed a weak binding affinity, following the order of Lip > Try > Pep > Amy. This sequence diverges from the inhibition efficacy determined by IC₅₀ values, suggesting that binding affinity alone is insufficient to predict the inhibition efficiency among different digestive enzymes. This discrepancy is attributed to various regulatory factors, including substrate competition and PA-induced conformational changes (Khaisaat et al., 2022). The Ka value of the same digestive enzyme increased with increasing DP, indicating that polydopamine with a higher DP has a stronger binding affinity for the digestive enzyme. This might explain the phenomenon that as the DP increases, the inhibitory effect of PAs on digestive enzymes becomes more pronounced.

The binding site number (n) for all PA-enzyme systems ranged from 0.2 to 1.5, deviating from the canonical single-site binding signature (n ≈ 1) (Martinez-Gonzalez et al., 2017), indicating heterogeneous multi-site interactions. Job's plots were employed to determine the binding stoichiometry (Huang, 1982), with the corresponding data summarized in Table S4. For F_1_, PA-to-enzyme binding ratios exhibited pronounced temperature dependence and instability across all enzyme systems. In contrast, higher-DP fractions (F_2_-F_8_) generally demonstrated more consistent stoichiometric behavior, likely due to their enhanced intermolecular interaction capacity. Try maintained a relatively stable stoichiometry centered at approximately 3:1 (PA: enzyme) under most conditions, whereas lipase predominantly interacted with PAs at ratios close to 4:3. Amy exhibited a narrow range of stoichiometric proportions, mostly between 3:2 and 5:3. Among the four enzymes, Pep displayed the widest stoichiometric fluctuation, with ratios varying from 1:1 to 3:1, depending on both the PA fraction and temperature. An integrated interpretation of the Job's plots and non-integer n values revealed that PAs with varying DP could bind to enzymes in a non-1:1 combination mode, further corroborating the heterogeneous multi-site binding characteristics.

The van't Hoff equation was utilized to determine thermodynamic parameters and elucidate the driving forces behind PA-enzyme binding (Martinez-Gonzalez et al., 2017; Tang et al., 2021). A comprehensive summary of all thermodynamic data is presented in Table 3. Classical biomolecular thermodynamics delineates three principal interaction modes: hydrogen bonding and van der Waals forces (ΔH < 0, ΔS < 0), hydrophobic interactions (ΔH > 0, ΔS > 0), and electrostatic interactions (ΔH ≈ 0, ΔS > 0) (Martinez-Gonzalez et al., 2017; Tang et al., 2021; Yang et al., 2025). A negative ΔG value signifies spontaneous complex formation, with a larger absolute ΔG indicating a stronger propensity for spontaneous binding (Liu et al., 2016; Wang et al., 2025). In this study, all supra-threshold PA-enzyme systems demonstrated negative ΔG, near-zero ΔH, and positive ΔS. These thermodynamic characteristics confirm that the binding of PAs with varying DP to enzymes is spontaneous, with electrostatic interactions serving as the primary force stabilizing the PA-enzyme complexes.

The formation of stable noncovalent complexes is consistent with the reversible inhibition mode identified through kinetic analysis. The increased binding affinity of high-DP PAs corresponds to their heightened surface electronegativity, further substantiating the predominant role of electrostatic attraction. The key DP-related thresholds and interaction characteristics are summarized in Table 4. To further elucidate the PA-mediated conformational changes in digestive enzymes, CD and FTIR spectroscopy were used to assess the alterations in the secondary structure of protein.

**Table 4:** Comparative overview of inhibitory modes, threshold characteristics, and key interaction parameters of four digestive enzymes in response to proanthocyanidins of different degrees of polymerization (F_1_-F_8_).

enzyme	Proanthocyanidins			
	inhibitory mode	fluorescence threshold	*λ*max threshold	main inferred interaction features
Pep	non-classical inhibition (DP = 1–12)	DP > 4	DP > 4	electrostatic interactions (DP > 4) and hydrogen bonding
Try	mixed-type inhibition (DP = 2–12)	DP > 2	DP > 5.5	electrostatic interactions (DP > 2) and hydrogen bonding
Lip	non-classical inhibition (DP = 1–12)	DP > 3	DP > 4	electrostatic interactions (DP > 3) and hydrogen bonding
Amy	non-classical inhibition (DP = 1–12)	DP > 2.5	DP > 12.5	electrostatic interactions (DP > 2.5) and hydrogen bonding

#### 3.6.2 CD analysis

Based on the multi-site synergistic binding behavior identified through kinetic and fluorescence analyses, circular dichroism (CD) spectroscopy was employed to assess PA-induced secondary structural changes in four digestive enzymes (Ianeselli et al., 2018; Rogers et al., 2019). CD measurements were conducted to observe enzyme conformational variations following incubation with F_1_-F_8_, with the relevant structural parameters detailed in Table S4. The addition of PA generally resulted in a decrease in the *α*-helix content of Pep, Try, Lip, and Amy by 15%, 8%, 14%, and 9%, respectively. Conversely, several individual PA fractions slightly increased the *α*-helix proportion of Try, Lip, and Amy by 7%, 3%, and 3%, respectively. The random coil content of all enzymes increased significantly by approximately 29%, 32%, 30%, and 33%. The extent of the structural rearrangement exhibited a nonlinear correlation with DP. CD results indicated that PA treatment transformed the ordered secondary structures of enzymes into more disordered conformations. This structural rearrangement contributed to the reduced enzymatic activity, consistent with previous studies on polyphenol-enzyme interactions (Martinez-Gonzalez et al., 2017).

#### 3.6.3 FTIR analysis

FTIR spectroscopy is a classical technique for characterizing biomolecular conformations and intermolecular interactions. In protein-ligand systems, amide I band-based FTIR spectra are widely used to monitor ligand-induced structural perturbations and to identify intermolecular forces (Yang et al., 2015; Yang et al., 2022). FTIR measurements were performed to complement the fluorescence and CD results, aiming to elucidate the intermolecular interactions and secondary structure changes of the PA-enzyme complex.

Distinct spectral changes were observed for all enzymes after PA supplementation (Fig. S19). The PA-Pep, PA-Try, and PA-Amy complexes exhibited broad absorption bands between 3700 and 2800 cm^−1^. According to previous studies on non-phenolic compounds, this broad region corresponds to O—H and N—H stretching vibrations, suggesting hydrogen bond formation between the PA hydroxyl groups and enzyme peptide chains (Kong & Yu, 2007; Zhang et al., 2022). Lip presented a distinct N—H stretching band near 3100 cm^−1^, reflecting an altered hydrogen-bonding microenvironment (Kong & Yu, 2007). Combined with the fluorescence findings, the FTIR results verified that hydrogen bonding and electrostatic interactions synergistically stabilized the PA-enzyme complexes.

FTIR spectroscopy was employed for the quantitative analysis of enzyme secondary structures, with the pertinent parameters detailed in Table S5. For Pep and Try, the *β*-sheet content decreased by 8–17% and 5–14%, respectively, while the *β*-turn content increased by 11–21% and 3–17%. In the case of Amy, PAs treatment resulted in simultaneous increases in the proportions of *β*-sheets (7–19%) and *β*-turns (11–25%). Lip exhibited varied structural responses: F_1_-F_3_ and F_7_ showed a reduction in *β*-sheet content by 5–38%, whereas F_4_-F_6_ and F_8_ experienced an increase in *β*-sheet content by 7–11%. Additionally, F_1_, F_3_-F_6_, and F_8_ exhibited a decrease in the *β*-turn content by 2–4%, whereas F_2_ and F_7_ showed an increase in this parameter by 5–16%. The primary conformational changes observed in the FTIR results included two scenarios: one involved a decrease in *β*-sheet structure content and an increase in *β*-turn content, while the other involved an increase in both *β*-sheet and *β*-turn contents. This indicates a complex structural reorganization between various digestive enzymes and PAs with varying DP. The decreased *β*-sheet content and increased *β*-turn content suggest the formation of a looser and more elastic conformation (Compton et al., 1987; Huang et al., 2008). Conversely, the increase in both *β*-sheet and *β*-turn contents indicates an increase in the compactness of the enzyme conformation (Yang et al., 2015; Yang et al., 2022).

#### 3.6.4 SEM analysis

SEM is a robust technique for morphological characterization, enabling the qualitative evaluation of the surface topography and the aggregation state of biomolecular complexes (Chen et al., 2025; Xu et al., 2021b). In this study, SEM was used to examine the microstructural features of PA-digestive enzyme complexes, facilitating the morphological interpretation of binding interactions and assessing the relationship between DP and aggregate compactness. Two representative PA fractions, low-DP F_2_ and high-DP F_8_, were selected to compare the microscopic morphologies of the corresponding PA-enzyme complexes.

Fig. 5 presents the results obtained from scanning electron microscopy, revealing notable differences in the microstructure between F_2_ and F_8_ and their complexes with digestive enzymes. F_2_ is characterized by a block-like structure with a uniform and porous surface, whereas F_8_ exhibits two distinct morphological features: a layered structure devoid of surface pores and rough, porous aggregates. Upon interaction with digestive enzymes, low-DP F_2_ develops a loose, porous network structure with a rough surface. In contrast, the aggregates formed by high-DP F_8_ were more compact, generally granular, and exhibited a marked reduction in porosity. These microstructural characteristics suggest that high-DP F_8_ exhibits stronger interactions with digestive enzymes, leading to greater aggregation and denser complex formation. This microscopic evidence supports distinct binding behaviors of the representative low-DP F_2_ and high-DP F_8_ fractions with digestive enzymes.

**Fig. 5:**
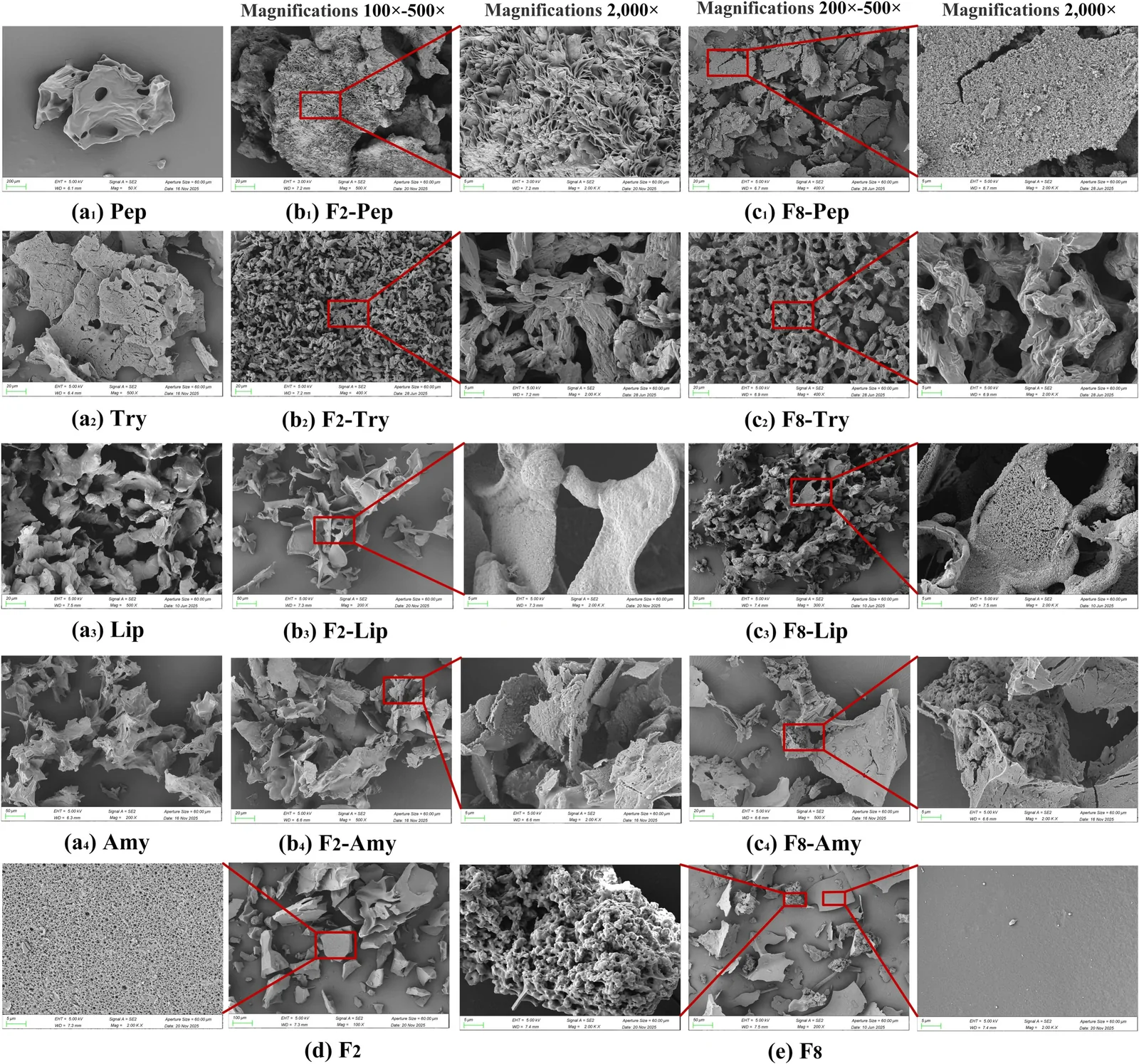
SEM micrographs of four digestive enzymes (pepsin (Pep), trypsin (Try), lipase (Lip), and *α*-amylase (Amy)): untreated digestive enzymes, digestive enzymes treated with proanthocyanidins (F_2_, F_8_), and proanthocyanidins (F_2_, F_8_) alone. Untreated digestive enzymes: Pep (a_1_), Try (a_2_), Lip (a_3_), and Amy (a_4_). Digestive enzymes treated with F_2_: Pep (b_1_), Try (b_2_), Lip (b_3_), and Amy (b_4_). Digestive enzymes treated with F_8_: Pep (c_1_), Try (c_2_), Lip (c_3_), and Amy (c_4_). F_2_ (d) and F_8_ (e).

The distinct morphological differences between F_2_ and F_8_ further elucidate the variation in their inhibitory effects on digestive enzymes. The high porosity of F_2_-digestive enzyme aggregates facilitated substrate diffusion and allowed easy access to enzyme active sites, thus explaining the weaker inhibitory potency of low-DP F_2_. The compact microstructure suggests that high-DP F_8_ possesses abundant binding moieties for extensive intermolecular association. This tight molecular stacking increases steric hindrance and restricts substrate accessibility, thereby enhancing the inhibitory efficiency of high-DP F_8_.

Overall, this study suggests that the inhibitory effects of PAs on digestive enzymes can be regulated by hierarchical and interrelated mechanisms associated with the molecular structure, binding mode, and aggregation behavior. These mechanistic interpretations are supported by complementary and mutually corroborative results from multiple experimental approaches, rather than relying on a single analytical method. Specifically, the PA-enzyme interaction represents a complex process in which the inhibitory potency may be jointly modulated by the DP, PA-to-enzyme concentration ratio, electrostatic and other noncovalent interactions, allosteric effects, and aggregate morphology.

This work highlights that DP may act as an important structural parameter governing PA-digestive enzyme interactions. Structural characterization indicated that an increased DP could correlate with an extended molecular conformation and greater exposure of hydroxyl groups. Inhibition assays for the four digestive enzymes revealed a positive correlation between the inhibitory effect and DP, with Try, Lip, and Amy being more susceptible than Pep. These findings suggest a clear structure-function relationship, whereby the inhibitory capacity of PA fractions progressively increases across the F_1_-F_8_ mDP gradient.

Furthermore, PA-enzyme interactions may follow concentration-dependent binding patterns. Enzyme kinetic analysis suggested that PAs could bind to both free enzymes and enzyme-substrate complexes, implying the coexistence of specific and multi-site binding modes. This inference is further supported by turbidity and fluorescence results: turbidity measurements indicate that nonlinear aggregation mainly occurs at higher PA-to-enzyme mass ratios (1:1 and 3:1) under elevated PA concentrations, while fluorescence analysis reveals static quenching behavior and non-1:1 binding stoichiometry. These consistent observations suggest that multi-site cooperative interactions become more dominant as the PA concentration increases. Meanwhile, PAs exhibit differential inhibitory modes toward the four enzymes via multi-site binding: mixed inhibition is observed for Try, whereas non-classical inhibition occurs for Pep, Lip, and Amy. Upon binding, PAs with different DP can induce enzyme conformational rearrangement, trigger static fluorescence quenching and synergistic binding effects, and present distinct DP-related threshold characteristics. When mDP exceeded the quenching threshold (Pep: 4; Try: 2; Lip: 3; Amy: 2.5), obvious intrinsic fluorescence quenching was observed. In contrast, PA fractions below the threshold (Pep: 4; Try: 5.5; Lip: 4; Amy: 12.5) may induce a blue shift in the maximum emission wavelength (*λ*max) of the enzymes.

This study also helps elucidate the link between PA binding, enzyme conformational changes, and aggregate formation. CD results showed that PA treatment may drive the transition of enzyme secondary structure toward random coils, implying conformational loosening and partial unfolding. FTIR analysis revealed changes in the β-sheet and β-turn contents of the PA-enzyme complexes, providing complementary information on PA-induced changes in enzyme secondary structure. Both fluorescence and FTIR spectra collectively suggest that electrostatic interactions and hydrogen bonding are the major driving forces for stabilizing PA-enzyme complexes.

Moreover, this work provides morphological evidence supporting a potential correlation between aggregation behavior and inhibitory performance. SEM observations revealed that high-DP F_8_-enzyme complexes tended to form tighter and more compact microstructures than their low-DP F_2_ counterparts. Integrating the structural, kinetic, spectroscopic, and morphological results, a plausible mechanistic model is proposed herein. At low concentrations, PAs may preferentially form simple oligomeric complexes with enzymes. At higher concentrations, multi-site electrostatic and hydrogen-bond crosslinking could further promote aggregate growth and maturation. Increased mDP may enhance the surface charge density and molecular chain length, which in turn facilitate the transition from loose porous aggregates to compact assemblies. This structural evolution may strengthen steric hindrance and limit substrate access to enzyme active sites, thereby potentially explaining the gradual enhancement of inhibitory activity with increasing DP. A comprehensive schematic diagram (Fig. 6) visually presents the dynamic mechanism of enzyme conformational changes and aggregation state transitions before and after the PA DP exceeds the critical threshold.

**Fig. 6:**
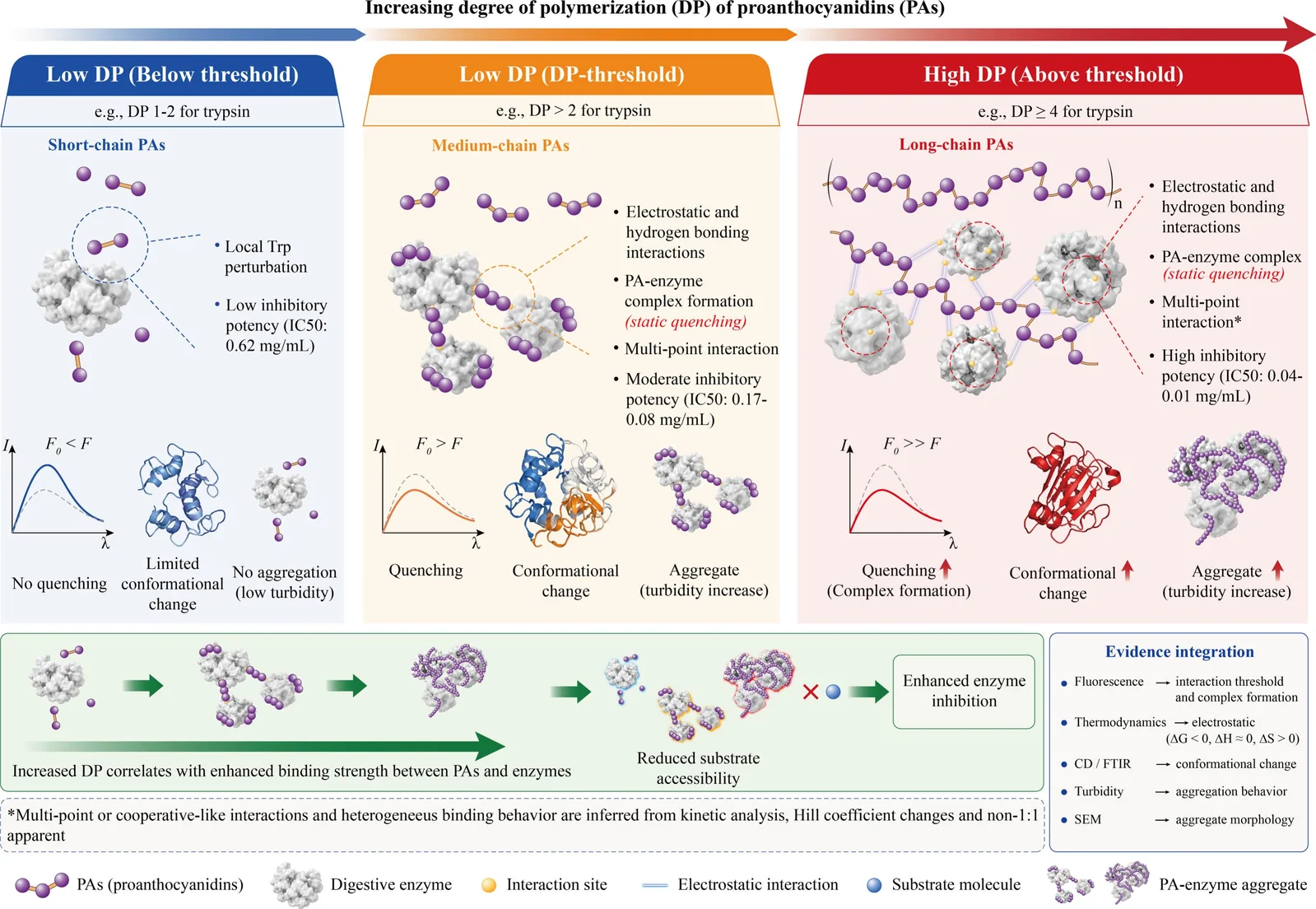
Mechanistic diagram of proanthocyanidin-induced enzyme conformational changes and aggregation behaviors across different degrees of polymerization (DP) thresholds.

While this study systematically elucidates the electrostatic interactions and aggregation characteristics between PAs and digestive enzymes in vitro, their physiological relevance requires further exploration. The gastrointestinal tract serves as the primary site for the biological function of dietary PAs, and its dynamic and complex microenvironment may significantly influence PA-enzyme interaction patterns and aggregate stability. Unlike the stable in vitro environment, the gastrointestinal lumen is a heterogeneous and dynamically changing system. Various coexisting factors, such as food matrix components, bile salts, variable pH, digestion time, and endogenous competitive substances, may collectively affect electrostatic binding and aggregation. The regulation of PA-enzyme complex formation and structural properties by these gastrointestinal factors remains to be clarified. Therefore, further research simulating realistic gastrointestinal conditions is imperative to elucidate these interactions and underlying mechanisms, thereby enhancing the mechanistic understanding of dietary PA functions within physiological contexts.

## 4 Conclusion

This study systematically elucidated the structure-activity relationship between the DP of PAs and their inhibitory effects on digestive enzymes, along with the underlying molecular interaction mechanisms. A consistent positive correlation between DP and inhibitory trends was observed across the four digestive enzymes, where an increased DP significantly enhanced the inhibitory efficacy of PAs. Mechanistically, compared to low-DP PAs, high-DP PAs exhibit extended molecular chains and increased surface electronegativity, facilitating robust multi-site binding with digestive enzymes through electrostatic forces and hydrogen bonds. These intermolecular interactions induced notable conformational rearrangements in the enzyme and promoted the formation of dense PA-enzyme aggregates. The compact aggregate architecture impeded substrate access to the enzyme active sites, thereby enhancing the inhibition. Furthermore, distinct enzyme-specific threshold responses to PA polymerization were identified, underscoring the selectivity of PA-enzyme interactions. Collectively, this study elucidates the intrinsic structure-function relationship of DP-regulated PA-inhibitory behaviors. These findings deepen the mechanistic understanding of PA-enzyme interactions and provide valuable theoretical insights.

## CRediT authorship contribution statement

**Chen Wang:** Writing – original draft, Visualization, Methodology, Investigation, Data curation, Conceptualization. **Qian Gao:** Writing – review & editing, Formal analysis. **Zhao Le:** Writing – review & editing, Software. **Peng Dai:** Writing – review & editing, Methodology. **Li Ma:** Writing – review & editing, Formal analysis. **Shuting Zhang:** Writing – review & editing, Supervision, Methodology, Formal analysis, Conceptualization. **Baoshan Sun:** Writing – review & editing, Supervision, Methodology, Funding acquisition, Conceptualization.

## Declaration of competing interest

The authors declare that they have no known competing financial interests or personal relationships that could have appeared to influence the work reported in this paper.

## Data Availability

Data will be made available on request.
