# Supplementary material for: Polymerization degree-driven impact on digestive enzyme inhibition by proanthocyanidins: mechanistic insights
Source: Food Chem X. 2026 Sep 25;39:104465. doi: 10.1016/j.fochx.2026.104465 (PMC13635492; doi:10.1016/j.fochx.2026.104465)
Supplement: Supplementary file 1 [file mmc1.docx]

**Supplementary material**

**Table S1.** The intensity of characteristic absorption band of the phenolic hydroxyl and aromatic ring skeleton vibration of proanthocyanidins with different degrees of polymerization (F_1_-F_8_)

|  | Intensity of absorption band  (3397 cm⁻¹) | Intensity of absorption band  (1625 cm⁻¹) | Intensity of absorption band  (1517 cm⁻¹) | Intensity of absorption band  (1461 cm⁻¹) |
| --- | --- | --- | --- | --- |
| F_1_ | 25764.49 | 1367.73 | 392.21 | 487.61 |
| F_2_ | 20679.14 | 1635.72 | 381.34 | 568.03 |
| F_3_ | 20438.74 | 1576.75 | 354.39 | 541.67 |
| F_4_ | 17969.57 | 1400.59 | 315.44 | 451.42 |
| F_5_ | 14081.26 | 1172.89 | 251.28 | 372.53 |
| F_6_ | 12916.44 | 989.42 | 244.16 | 346.59 |
| F_7_ | 7967.89 | 627.71 | 104.17 | 222.83 |
| F_8_ | 2971.95 | 324.16 | 74.29 | 97.18 |

**Table S2.** Hill coefficients (nH) of pepsin (Pep), trypsin (Try), lipase (Lip) and *α*-amylase (Amy) for proanthocyanidins with different degrees of polymerization (F_1_-F_8_)

|  | Concentration 1 | Concentration 2 | Concentration 3 | Concentration 4 | Concentration 5 | Concentration 6 |
| --- | --- | --- | --- | --- | --- | --- |
| F_1_-Pep | 1.366 | 1.733 | 1.553 | 1.692 | 1.042 | 1.274 |
| F_3_-Pep | 1.12 | 1.141 | 1.558 | 0.9996 | 1.373 | 2.447 |
| F_5_-Pep | 1.325 | 1.364 | 1.505 | 1.466 | 1.578 | 1.379 |
| F_7_-Pep | 1.201 | 1.247 | 1.572 | 1.899 | 1.008 | 1.938 |
| F_8_-Pep | 1.136 | 1.286 | 1.394 | 1.407 | 1.589 | 1.614 |
| F_1_-Try | 1.165 | 1.003 | 1.003 | 1.056 | 1.035 | 1.007 |
| F_3_-Try | 1.039 | 1.053 | 1.056 | 1.066 | 1.068 | 1.078 |
| F_5_-Try | 1.037 | 1.015 | 1.027 | 1.016 | 1.042 | 1.024 |
| F_7_-Try | 0.9769 | 0.9429 | 0.9626 | 0.9246 | 0.9263 | 0.9634 |
| F_8_-Try | 1.023 | 1.032 | 1.049 | 1.04 | 1.047 | 1.015 |
| F_1_-Lip | 1.512 | 1.152 | 1.155 | 1.289 | 1.135 | 1.093 |
| F_3_-Lip | 1.26 | 1.136 | 1.299 | 1.067 | 1.048 | 1.339 |
| F_5_-Lip | 1.346 | 1.179 | 1.737 | 1.617 | 1.475 | 1.344 |
| F_7_-Lip | 1.149 | 0.9266 | 1.188 | 1.095 | 1.122 | 1.205 |
| F_8_-Lip | 1.119 | 1.207 | 1.043 | 0.9586 | 1.134 | 1.043 |
| F_1_-Amy | 1.115 | 1.186 | 1.162 | 1.198 | 1.208 | 1.195 |
| F_3_-Amy | 1.146 | 1.106 | 1.19 | 1.015 | 1.105 | 1.162 |
| F_5_-Amy | 1.081 | 1.097 | 1.155 | 1.144 | 1.184 | 1.133 |
| F_7_-Amy | 1.157 | 1.268 | 1.139 | 1.348 | 1.381 | 1.364 |
| F_8_-Amy | 1.12 | 1.094 | 1.061 | 1.084 | 1.019 | 1.021 |

Abbreviations: Concentration 1 to Concentration 6 denotes the progression from low to high concentrations of proanthocyanidins with different degrees of polymerization

**Table S3.** Binding equivalents of proanthocyanidins with different degrees of polymerization (F_1_-F_8_) to pepsin (Pep), trypsin (Try), lipase (Lip), and *α*-amylase (Amy)

|  | Fx:Pep | | |  | Fx:Try | | |  | Fx:Lip | | |  | Fx:Amy | | |
| --- | --- | --- | --- | --- | --- | --- | --- | --- | --- | --- | --- | --- | --- | --- | --- |
|  | 37 ℃ | 25 ℃ | 18 ℃ |  | 37 ℃ | 25 ℃ | 18 ℃ |  | 37 ℃ | 25 ℃ | 18 ℃ |  | 37 ℃ | 25 ℃ | 18 ℃ |
| F_1_ | 5:3 | 3:2 | 3:2 |  | 3:1 | 5:2 | 3:1 |  | 5:4 | 5:4 | 3:2 |  | 3:2 | 3:2 | 3:2 |
| F_2_ | 2:1 | 2:1 | 2:1 |  | 3:1 | 5:2 | 5:3 |  | 3:2 | 2:1 | 5:3 |  | 5:3 | 5:3 | 5:2 |
| F_3_ | 3:2 | 2:1 | 5:3 |  | 3:1 | 3:1 | 3:1 |  | 5:2 | 5:4 | 1:1 |  | 2:1 | 1:1 | 5:2 |
| F_4_ | 5:2 | 5:3 | 5:3 |  | 3:1 | 3:1 | 3:1 |  | 4:3 | 5:4 | 5:4 |  | 2:1 | 5:3 | 5:3 |
| F_5_ | 2:1 | 5:3 | 3:2 |  | 5:2 | 3:1 | 3:1 |  | 4:3 | 1:1 | 5:4 |  | 3:2 | 3:2 | 3:2 |
| F_6_ | 1:1 | 4:3 | 2:1 |  | 3:1 | 5:2 | 3:1 |  | 3:4 | 1:1 | 1:1 |  | 3:2 | 3:2 | 3:2 |
| F_7_ | 1:1 | 4:3 | 5:3 |  | 3:1 | 3:1 | 3:1 |  | 5:4 | 5:4 | 1:1 |  | 3:2 | 3:2 | 3:2 |
| F_8_ | 3:1 | 4:3 | 5:3 |  | 4:5 | 7:2 | 4:3 |  | 4:3 | 4:3 | 4:3 |  | 3:2 | 3:2 | 3:2 |

**Table S4.** Secondary structure composition of pepsin (Pep), trypsin (Try), lipase (Lip), and *α*-amylase (Amy) in the presence and absence of proanthocyanidins with different degrees of polymerization (F_1_-F_8_) determined by CD spectroscopy.

| Sample | *α*-helix (%) | *β*-sheet (%) | *β*-turn (%) | Random coil (%) |
| --- | --- | --- | --- | --- |
| Pep | 15.17±0.93^a^ | 49.77±1.38^a^ | 18.97±2.91^d^ | 16.57±0.98^d^ |
| F_1_-Pep | 1.93±0.60^b^ | 45.50±3.08^ab^ | 21.43±1.72^bcd^ | 28.53±5.69^cd^ |
| F_2_-Pep | -1.70±3.41^b^ | 35.60±4.33^bc^ | 19.97±1.46^cd^ | 40.13±12.52^bc^ |
| F_3_-Pep | -3.07±0.83^b^ | 39.97±6.99^abc^ | 21.00±0.70^bcd^ | 39.03±7.93^bc^ |
| F_4_-Pep | -0.23±2.80^b^ | 45.77±4.05^ab^ | 21.17±0.78^bcd^ | 29.57±6.17^cd^ |
| F_5_-Pep | -3.13±2.53^b^ | 35.10±4.86^bc^ | 19.63±1.92^cd^ | 45.30±4.90^ab^ |
| F_6_-Pep | -2.10±2.52^b^ | 27.63±17.29^c^ | 24.07±3.46^bc^ | 44.30±13.88^ab^ |
| F_7_-Pep | -0.10±5.55^b^ | 48.00±0.98^ab^ | 23.30±1.77^b^ | 24.67±1.88^d^ |
| F_8_-Pep | -3.60±6.54^b^ | 14.47±4.83^d^ | 30.00±3.04^a^ | 37.23±3.27^a^ |
| Try | 4.83±3.52^b^ | 45.63±1.46^a^ | 18.73±5.81^b^ | 24.23±3.02^b^ |
| F_1_-Try | -1.85±0.64^cd^ | 43.20±8.06^ab^ | 19.20±0.71^b^ | 31.55±0.07^b^ |
| F_2_-Try | -3.30±2.59^d^ | 49.60±2.07^a^ | 22.77±1.00^b^ | 30.97±2.75^b^ |
| F_3_-Try | 0.47±0.76^bcd^ | 49.37±0.49^a^ | 22.33±0.15^b^ | 26.27±0.58^b^ |
| F_4_-Try | 1.37±1.63^cd^ | 45.23±5.69^a^ | 20.63±2.73^b^ | 31.67±6.49^b^ |
| F_5_-Try | -3.57±2.78^d^ | 35.40±6.62^b^ | 19.23±1.80^b^ | 46.60±11.00^a^ |
| F_6_-Try | -2.50±2.18^cd^ | 49.27±0.40^a^ | 22.07±0.50^b^ | 27.57±2.67^b^ |
| F_7_-Try | 11.03±7.56^a^ | 44.43±8.88^ab^ | 18.40±6.38^b^ | 26.00±4.48^b^ |
| F_8_-Try | 3.67±1.30^bc^ | 23.13±1.53^c^ | 29.73±0.91^a^ | 36.95±1.91^a^ |
| Lip | 9.27±13.32^ab^ | 50.90±19.28^a^ | 16.43±4.07^c^ | 21.77±7.73^b^ |
| F_1_-Lip | 12.80±3.99^a^ | 45.63±2.27^a^ | 16.77±3.91^c^ | 24.30±1.41^b^ |
| F_2_-Lip | -5.17±3.52^b^ | 16.97±4.41^b^ | 29.63±2.64^a^ | 50.13±6.55^a^ |
| F_3_-Lip | 7.20±3.25^ab^ | 16.40±1.98^b^ | 25.30±0.57^abc^ | 51.35±1.91^a^ |
| F_4_-Lip | 6.17±3.81^ab^ | 49.67±0.86^a^ | 20.97±1.42^abc^ | 22.00±3.15^b^ |
| F_5_-Lip | 6.83±13.31^ab^ | 19.17±9.10^b^ | 27.13±4.46^ab^ | 37.93±8.60^a^ |
| F_6_-Lip | 2.50±0.40^ab^ | 48.13±0.12^a^ | 22.73±0.35^abc^ | 25.30±0.72^b^ |
| F_7_-Lip | 4.10±9.64^ab^ | 25.63±20.84^b^ | 25.23±4.59^abc^ | 43.67±15.48^a^ |
| F_8_-Lip | 10.93±7.58^ab^ | 46.77±1.97^a^ | 18.17±4.50^bc^ | 23.67±1.35^b^ |
| Amy | 10.20±6.56^a^ | 46.97±20.17^a^ | 18.07±5.46^c^ | 23.63±0.60^b^ |
| F_1_-Amy | 1.37±6.76^a^ | 16.67±6.77^c^ | 29.73±5.39^a^ | 47.87±10.09^a^ |
| F_2_-Amy | 12.43±6.70^a^ | 49.07±0.80^a^ | 18.37±1.85^c^ | 19.80±5.97^b^ |
| F_3_-Amy | 6.00±5.20^a^ | 46.63±1.93^a^ | 20.00±4.53^c^ | 26.73±1.39^b^ |
| F_4_-Amy | 2.83±2.30^a^ | 18.80±3.03^c^ | 26.60±2.31^ab^ | 50.13±2.66^a^ |
| F_5_-Amy | 3.07±1.38^a^ | 44.70±5.43^a^ | 21.77±0.49^bc^ | 24.63±1.29^b^ |
| F_6_-Amy | 1.97±2.82^a^ | 12.17±4.27^c^ | 27.13±0.58^ab^ | 56.53±6.97^a^ |
| F_7_-Amy | 2.30±2.55^a^ | 23.83±19.48^bc^ | 23.87±3.69^abc^ | 43.87±17.53^a^ |
| F_8_-Amy | 10.60±13.57^a^ | 35.50±15.24^ab^ | 19.70±3.41^c^ | 47.50±6.44^b^ |

Abbreviations: For values within the same column, significance analysis indicated by different letters is limited to comparisons among F_1_-F_8_ fractions for the same digestive enzyme. Different lowercase letters represent significant differences (*p* < 0.05).

**Table S5.** Secondary structure composition of pepsin (Pep), trypsin (Try), lipase (Lip), and *α*-amylase (Amy) in the presence and absence of proanthocyanidins with different degrees of polymerization (F_1_-F_8_) determined by FTIR spectroscopy

| Sample | *α*-helix (%) | *β*-sheet (%) | *β*-turn (%) | Random coil (%) |
| --- | --- | --- | --- | --- |
| Pep | 8.37 | 57.57 | 21.44 | 12.62 |
| F_1_-Pep | 12.05 | 42.52 | 32.54 | 12.89 |
| F_2_-Pep | 0.00 | 45.01 | 32.55 | 22.44 |
| F_3_-Pep | 9.40 | 40.36 | 40.85 | 9.39 |
| F_4_-Pep | 4.83 | 43.41 | 42.46 | 9.30 |
| F_5_-Pep | 0.00 | 45.20 | 41.63 | 13.18 |
| F_6_-Pep | 0.00 | 49.19 | 38.44 | 12.36 |
| F_7_-Pep | 8.69 | 49.32 | 32.78 | 9.21 |
| F_8_-Pep | 9.91 | 45.86 | 33.96 | 10.27 |
| Try | 9.12 | 53.36 | 26.16 | 11.36 |
| F_1_-Try | 10.51 | 48.49 | 29.49 | 11.51 |
| F_2_-Try | 11.62 | 45.22 | 30.11 | 13.04 |
| F_3_-Try | 24.43 | 42.39 | 33.18 | 0.00 |
| F_4_-Try | 23.87 | 42.77 | 33.36 | 0.00 |
| F_5_-Try | 0.00 | 42.97 | 43.52 | 13.52 |
| F_6_-Try | 14.34 | 44.87 | 25.86 | 14.93 |
| F_7_-Try | 9.43 | 39.88 | 41.19 | 9.50 |
| F_8_-Try | 11.07 | 39.09 | 39.35 | 10.49 |
| Lip | 8.36 | 64.37 | 17.64 | 10.21 |
| F_1_-Lip | 28.21 | 58.41 | 13.39 | 0.00 |
| F_2_-Lip | 20.84 | 26.46 | 33.88 | 18.82 |
| F_3_-Lip | 28.88 | 57.50 | 13.62 | 0.00 |
| F_4_-Lip | 0.00 | 72.86 | 16.09 | 11.56 |
| F_5_-Lip | 0.00 | 74.10 | 14.63 | 11.27 |
| F_6_-Lip | 0.00 | 71.82 | 15.93 | 12.25 |
| F_7_-Lip | 0.00 | 59.00 | 22.61 | 18.39 |
| F_8_-Lip | 0.00 | 75.34 | 14.76 | 10.35 |
| Amy | 23.66 | 28.86 | 24.86 | 22.62 |
| F_1_-Amy | 10.63 | 38.48 | 36.18 | 14.70 |
| F_2_-Amy | 9.32 | 40.24 | 41.07 | 9.36 |
| F_3_-Amy | 9.70 | 41.97 | 38.49 | 9.84 |
| F_4_-Amy | 6.49 | 35.95 | 49.98 | 7.58 |
| F_5_-Amy | 11.55 | 46.33 | 42.12 | 0.00 |
| F_6_-Amy | 9.46 | 40.79 | 39.84 | 9.91 |
| F_7_-Amy | 8.86 | 41.71 | 40.21 | 9.22 |
| F_8_-Amy | 0.00 | 47.91 | 40.39 | 11.70 |

**Figure S1.** HSCCC chromatogram on the separation of grape seed proanthocyanidins using head-tail elution modes. Their elution corresponded to the degrees of polymerization: F_1_, monomeric proanthocyanidins; F_2_–F_5_, oligomeric proanthocyanidins; F_6_-F_8_, polymeric proanthocyanidins.

**Figure S2.** UPLC chromatograms of phloroglucinolysis of fractions F_1_-F_8_ obtained by HSCCC separation of grape seed extract.

**Figure S3.** The profiles of initial enzymatic reaction rates plotted against concentrations of pepsin (Pep) in the presence of proanthocyanidin fractions with different degrees of polymerization (F_1_-F_8_) at various concentrations.

**Figure S4.** The profiles of initial enzymatic reaction rates plotted against concentrations of trypsin (Try) in the presence of proanthocyanidin fractions with different degrees of polymerization (F_1_-F_8_) at various concentrations.

**Figure S5.** The profiles of initial enzymatic reaction rates plotted against concentrations of lipase (Lip) in the presence of proanthocyanidin fractions with different degrees of polymerization (F_1_-F_8_) at various concentrations.

**Figure S6.** The profiles of initial enzymatic reaction rates plotted against concentrations of *α*-amylase (Amy) in the presence of proanthocyanidin fractions with different degrees of polymerization (F_1_-F_8_) at various concentrations.

**Figure S7.** Fluorescence emission spectra of pepsin in the presence of proanthocyanidins with different degrees of polymerization (F_1_-F_8_) at various concentrations at 291, 298, and 310 K.

(Note: c (F_1_-F_8_)= 0, 0.06, 0.09, 0.12, 0.15, and 0.18 *μ*g/mL for curves a→f, respectively)

**Figure S8.** Fluorescence emission spectra of trypsin in the presence of proanthocyanidins with different degrees of polymerization (F_1_-F_8_) at various concentrations at 291, 298, and 310 K.

(Note: c (F_1_-F_8_)= 0, 0.06, 0.09, 0.12, 0.15, and 0.18 *μ*g/mL for curves a→f, respectively)

**Figure S9.** Fluorescence emission spectra of lipase in the presence of proanthocyanidins with different degrees of polymerization (F_1_-F_8_) at various concentrations at 291, 298, and 310 K.

(Note: c (F_1_-F_8_)= 0, 0.06, 0.09, 0.12, 0.15, and 0.18 *μ*g/mL for curves a→f, respectively)

**Figure S10.** Fluorescence emission spectra of *α*-amylase in the presence of proanthocyanidins with different degrees of polymerization (F_1_-F_8_) at various concentrations at 291, 298, and 310 K.

(Note: c (F_1_-F_8_)= 0, 0.06, 0.09, 0.12, 0.15, and 0.18 *μ*g/mL for curves a→f, respectively)

**Figure S11.** Stern-Volmer plots describing fluorescence quenching of pepsin (a - h) in the presence of proanthocyanidins with different degrees of polymerization (F_1_-F_8_) at 291, 298, and 310 K.

**Figure S12.** Stern-Volmer plots describing fluorescence quenching of trypsin (a - h) in the presence of proanthocyanidins with different degrees of polymerization (F_1_-F_8_) at 291, 298, and 310 K.

**Figure S13.** Stern-Volmer plots describing fluorescence quenching of lipase (a - h) in the presence of proanthocyanidins with different degrees of polymerization (F_1_-F_8_) at 291, 298, and 310 K.

**Figure S14.** Stern-Volmer plots describing fluorescence quenching of *α*-amylase (a - h) in the presence of proanthocyanidins with different degrees of polymerization (F_1_-F_8_) at 291, 298, and 310 K.

**Figure S15.** The double-logarithmic Stern-Volmer plots for the static quenching of pepsin (a - d) by proanthocyanidins with different degrees of polymerization (F_5_-F_8_) at 291, 298, and 310 K.

**Figure S16.** The double-logarithmic Stern-Volmer plots for the static quenching of trypsin (a - g) by proanthocyanidins with different degrees of polymerization (F_2_-F_8_) at 291, 298, and 310 K.

**Figure S17.** The double-logarithmic Stern-Volmer plots for the static quenching of lipase (a - g) by proanthocyanidins with different degrees of polymerization (F_2_-F_8_) at 291, 298, and 310 K.

**Figure S18.** The double-logarithmic Stern-Volmer plots for the static quenching of *α*-amylase (a - f) by proanthocyanidins with different degrees of polymerization (F_3_-F_8_) at 291, 298, and 310 K.

**Figure S19.** FTIR spectra of pepsin (Pep), trypsin (Try), lipase (Lip), and *α*-amylase (Amy) in the presence of proanthocyanidins with different degrees of polymerization (F_1_-F_8_).
